# Placental malaria: a systematic review and meta-analysis of global burden, risk factors, and maternal and foetal outcomes

**DOI:** 10.7189/jogh.15.04355

**Published:** 2025-11-28

**Authors:** Sonia Menon, Flavia D’Alessio, Nita Chaudhuri, Chukwuemeka Onwuchekwa, Mandeep Kaur, Irene Nkumama, Ole F Olesen

**Affiliations:** 1 Epitech research, Auderghem, Belgium; 2 European Vaccine Initiative, Universitäts Klinikum Heidelberg, Heidelberg, Germany; 3 Saffron Global Health, Croissy sur Seine, France; 4 P-95, Koning Leopold III-laan 1, 3001 Leuven

## Abstract

**Background:**

Pregnant women in malaria-endemic countries are at risk of placental malaria (PM), which can lead to adverse outcomes for both mothers and children. Histology of placental tissue is the gold standard for diagnosing PM, as it can detect current and past infections. Prior reviews focussed on malaria in pregnancy generally; in this systematic review, we specifically examine PM due to *Plasmodium falciparum*, its associated risk factors, and its impact on maternal and foetal outcomes.

**Methods:**

We included studies performed since 2013, reflecting important updates in WHO policy recommendations for PM control efforts and resistance to sufadoxine-primethamine resistance over the past decade. After extracting relevant data, we calculated the pooled prevalence, odds ratios (ORs), and risk ratios. We assessed the quality of the included studies using the Newcastle-Ottawa scale.

**Results:**

The review included 50 studies, 45 of which were from sub-Saharan Africa (SSA), with 15 (33%) of them using histological diagnosis. Global PM prevalence was 17% (95% confidence interval (CI) = 12–21), rising to 23% (95% CI = 1–4) in histology-based studies. Prevalence was higher in SSA (19%; 95% CI = 14–24) than in other regions (4%; 95% CI = 1–9), with West Africa showing the highest rates. One study including only HIV-positive women reported a PM prevalence of 45% (95% CI = 38–52) compared to 17% (95% CI = 10–25) in HIV-negative women. One study on stillbirth showed an OR of 3.81 (95% CI = 1.22–11.94) and primigravidae had pooled ORs of 1.61 (95% CI = 0.91–2.84) compared to multigravidae. The ORs and CIs for congenital malaria, malaria in infancy, preterm birth, and low birth weight were wide, indicating imprecision.

**Conclusions:**

Our meta-analysis reveals a high PM burden in high- malaria transmission areas, especially among primigravidae and HIV-positive women. We note that PM remains high in SSA, with regional variation, with one in four pregnant women diagnosed by histological examination of the placenta, reflecting both current and past PM exposure. Reliance on non-histological methods may lead to underestimation of true PM prevalence. Due to wide confidence intervals and limited data, we could draw no conclusions on the impact of PM on maternal and foetal outcomes. Residual high heterogeneity reflects real-world diversity across populations, strengthening the generalisability of our findings.

Pregnancy-associated malaria is a significant public health issue globally, but especially in sub-Saharan Africa (SSA), where it contributes to an estimated 75 000–200 000 infant deaths annually [1]. It results from *Plasmodium *infection during pregnancy, particularly *P. falciparum*, and can lead to placental malaria (PM). This condition is marked by sequestration of infected erythrocytes in the placenta *via* VAR2CSA, a parasite protein that binds to chondroitin sulfate A (CSA) on placental tissue [2]. This accumulation disrupts the maternal-foetal exchange and triggers inflammation, leading to maternal morbidity and adverse foetal outcomes [2]. Histological analysis is considered the gold standard for PM diagnosis due to its ability to detect these specific pathological changes, and it allows the detection of past infection in the placenta during the pregnancy, in contrast to most alternative methods [3].

Aside from the SSA [4], PM also occurs in parts of Asia [5] and Latin America [6]. It may lead to various types of poor pregnancy outcomes [7], affecting both the mother and foetus. Previous research has reported higher rates of maternal anaemia [8] and perinatal outcomes such as preterm birth [7], stillbirth [9], small gestational age [8], and low birth weight (LBW) [10], with LBW being a strong predictor of neonatal as well as lifelong health adverse outcomes [11].

Studies have notably shown that primigravidae have a higher risk of developing PM compared to multigravidae [12]. In regions with low transmission rates, however, all pregnancies are at heightened risk of PM due to the overall lower immunity against malaria in the population [13]. Co-infections with malaria and HIV worsen morbidity and mortality for each disease [14], constituting another potential risk factor for PM. HIV-positive pregnant women were shown to have an increased risk of malaria-associated adverse pregnancy outcomes [15].

For prevention of malaria in pregnancy, the World Health Organization (WHO) recommends the use of insecticide-treated bed nets (ITNs), intermittent preventive treatment with sulfadoxine-pyrimethamine (IPTp-SP), prompt diagnosis, and effective case management [16]. However, many pregnant women remain unprotected due to several limitations in current control measures. First, IPTp-SP starts only at 13 weeks of gestation, as it may be teratogenic during the first trimester [17]. Furthermore, while the use of SP in SSA for clinical malaria [18] was stopped due to emerging resistance, it is still recommended for IPTp in areas of moderate-to-high malaria transmission in Africa [19]. Second, the utilisation of ITNs among pregnant women in SSA remains suboptimal [20]. Complementary prophylactic measures such as a general malaria vaccine that prevents blood-stage infection during pregnancy, or a PM-specific vaccine would, therefore, constitute important tools for reducing PM morbidity and mortality. Two VAR2CSA-derived PM vaccine candidates – PRIMVAC and PAMVAC – have been recently tested in clinical trials. Both were found to be safe, immunogenic, and to induce good functional anti-adhesion antibodies against the homologous parasite strain [21,22].

To gauge the current global burden of PM caused by *P. falciparum* and to capture the current landscape of control policies, interventions, and the emergence of drug resistance to SP over the past decade, we reviewed eligible studies performed between 2013–24 and pooled the prevalence of PM in this period, along with the effect measures for risk factors and their associations with maternal and foetal outcomes.

## METHODS

We registered this review in PROSPERO (CRD42024540146), performed it according to the Cochrane guidance on systematic reviews, and reported our findings it in accordance with the PRISMA guidelines [23].

### Inclusion and exclusion criteria

We included all peer-reviewed original studies reporting on the prevalence of PM, with its attendant risk factors, along with maternal (anaemia, cerebral anaemia, severe malaria, and mortality) and foetal outcomes (abortion, stillbirth, preterm delivery, LBW, small for gestational age, neonatal mortality, congenital malaria, infant mortality, and infant anaemia), provided they presented clear attribution to *P. falciparum* infection, either directly or by species predominance. Studies with standard care (IPTp-SP) control arms were included; those testing dihydroartemisinin-piperaquine or reporting only immunological endpoints were excluded. We selected 2013 as the cut-off for study inclusion to align with the introduction of significant updates in the WHO IPTp SP policy [24], allowing for a contemporary and relevant assessment of PM global burden, risk factors, and outcome. outcome. Therefore, we also excluded studies spanning pre- and post-2013, unless data were disaggregated. We did not consider non-original studies and conference abstracts.

### Search strategy, identification, and extraction of studies

We searched PubMed (23 March 2025), Embase (23 March 2025), Scopus (24 March 2025), and the Malaria in Pregnancy Library (18 March 2025), using MeSH terms and keywords related to *P. falciparum*, PM, and pregnancy outcomes (**Online Supplementary Document**). All citations were imported into Rayyan software (Rayyan, Cambridge, Massachusetts, USA). After deduplication, two authors (NC, SM) independently screened titles, reviewed full texts, checked reference lists, and extracted data on study characteristics, demographics, PM cases, risk factors, and outcomes. Discrepancies were resolved by consensus.

### Risk of bias assessment

Two authors (SM and NC) independently assessed study quality using an adapted Newcastle-Ottawa Scale, evaluating representativeness, sample size, and diagnostic accuracy. Scores ranged up to 5 for cross-sectional and 6 for cohort or case-control studies. Discrepancies in the rating process were resolved by consensus (**Online Supplementary Document**).

### Statistical synthesis

We performed the meta-analysis in Stata, version 18 (StataCorp LLC, College Station, Texas, USA), utilising its built-in meta-analysis functions. We calculated PM prevalence as the proportion of pregnant women diagnosed with PM in each study, with pooled estimates generated using the ‘metaprop’ command, which uses the binomial distribution for modelling proportions and implements variance-stabilising transformations. The default transformation, Freeman-Tukey double arcsine transformation [25] was used to stabilise the variance of prevalence estimates. To examine associations between PM, risk factors, and foetal outcomes, we conducted a meta-analysis of odds ratios (ORs) or risk ratios (RRs) alongside their 95% confidence intervals (CI) for precision, comparing outcomes in women or offspring with PM to those without. We did not assess publication bias, as no outcome was reported by ten or more studies [26].

To account for potential heterogeneity amongst the trials, we applied random-effects models using restricted maximum-likelihood method [27]. Assessment of heterogeneity within and between study groups was performed using Cochran’s Q test, with a significance level of *P* < 0.1 suggesting the presence of statistically significant heterogeneity [26]. We used the *I*^2^ statistic to estimate the percentage of observed between-study variability due to between-study heterogeneity, as opposed to within study sampling error [28]. Roughly, this statistic ranges from 0 to 100%, with values of 0–40%, 30–60%, 50–90%, and 75–100% representing low, moderate, substantial, and considerable heterogeneity, respectively [26].

We conducted subgroup analyses by diagnostic method (histology *vs*. microscopy/PCR), geographic setting (SSA *vs*. non-SSA, by region and country), HIV status (HIV+, HIV−, mixed/unspecified), and sample representativeness. We also stratified by transmission setting (high, not reported, low) as reported by authors. For studies without specified transmission levels, sensitivity analyses reclassified them as high or low transmission

## RESULTS

### Study, participant characteristics, and risk of bias assessment

After screening 4327 abstracts, we excluded 4128 records and retained 199 for full text assessment. We then excluded 150 records, leaving 50 studies reported in 49 papers in the review. These studies were conducted in 17 countries: 14 in SSA [13,29–69], two in Southeast Asia [70,71] and one in Latin America [6,72,73] (Figure S1 in the **Online Supplementary Document**). In total, 21 882 pregnant women provided data for our pooled PM prevalence. The sample size per study, when reported, ranged from 33 to 2384, while the mean age of study participants, when provided, was between 21.06 (standard deviation (SD) = 3.03) and 31.9 (SD = 5.3) years (**Table 1**)

**Table 1:** Characteristics of included studies

Author, year	Pregnant women	Country	Study design	Dates	Transmission	Age, x̄ (SD)	Primigravid (%)	HIV	ITNs, %	IPTp-SP, %	Diagnosis	NOS score
Alemayehu, 2024 [29]	180	Ethiopia	Cross Sectional	2022–23	NR	26 (18-42)	24,9	Mixed or not specified	73		Placental smear microscopy	3
Anchang-Kimbi, 2020 [30]	465	Cameroon	Cross Sectional	2016–17	High	25.82 (5.5)	30	Mixed or not specified	68	100	Placental histology	4
Ahmed, 2023 [31]	2384	Sudan	Cross Sectional	2020–20	High	NR (NR)	NR	Mixed or not specified			Placental smear microscopy	3
Akafa, 2014 [32]	108	Nigeria	Prospective Cohort	NR (most likely from 2013 onwards)	High	27.75 (NR)	35	Mixed or not specified		100	Placental histology	4
Andronescu, 2022 [33]	343	Uganda	Cross Sectional	2017–19	High	24.6 (6.1)	70	Only HIV negative	38	50	Placental histology	4
Babalola, 2017 [34]	211	Nigeria	Cross Sectional	2014	High	NR (NR)	32	Mixed or not specified	60	78	Placental smear microscopy	4
Braun, 2015 [35]	676	Uganda	Cross Sectional	2013	High	25 (range = 18–42)*	22	Mixed or not specified	65	92	Placental smear microscopy	2
Briand, 2016 [70]	331	Laos	Cross Sectional	2014	High	25 (NR)	67	Mixed or not specified	92		Placental blood RT-PCR	2
Cardona-Arias, 2023 [74]	549	Colombia	Cross Sectional	2016–19	High	NR (NR)	NR	Mixed or not specified	NR	NR	Placental blood RT-PCR	2
Cardona-Arias, 2024 [72]	431	Colombia	Prospective Cohort	2020	NR	Range = 14–24	79	Mixed or not specified	NR	NR	Placental blood RT-PCR	5
Djontu, 2016 [36]	108	Cameroon	Cross Sectional	2013–15	NR	25 (range = 16–37)*	36	Mixed or not specified			Placental histology	2
Dosoo, 2021 [37]	1642	Ghana	Cross Sectional	2017–19	NR	27.69 (6.01)	23	Only HIV negative	65		Placental histology	4
Douamba, 2014 [38]	238	Burkina Faso	Cross Sectional	2013–14	High	26.46 (4.96)	NR	Mixed or not specified	87	85	Placental smear microscopy	2
Emechebe, 2022 [39]	440	Nigeria	Prospective Cohort	2018–19	High	28.45 (3.06)	43	Only HIV negative			Placental histology	6
Epuitai, 2024 [40]	366	Uganda	Cross Sectional	2018–19	High	25.34 (5.73)	33,9	Mixed or not specified	86,2	91,1	Rapid diagnostic test	2
Felician, 2022 [41]	80	Tanzania	Cross Sectional	2016	NR	32 (range = 18–40)*	43	Mixed or not specified			Placental histology	3
Hangi, 2019 [42]	54	Uganda	Cross Sectional	2014	High	NR (NR)	NR	Mixed or not specified			Placental smear microscopy	1
Idih, 2016 [75]	180	Nigeria	Cross Sectional	2013–14	NR	27.1 (5.4)	NR	Mixed or not specified	63	58	Placental smear microscopy	1
Kajubi, 2019 [43]	322	Uganda	IPT-sp arm of RCT	2016–17	High	NR (NR)	NR	Only HIV negative			Placental smear microscopy	2
Kamga, 2024 [44]	380	Cameroon	Cross Sectional	2021	High	Range ≥16	38	Only HIV negative	48	88	Placental blood RT-PCR	3
Limenih, 2021 [45]	218	Ethiopia	Cross Sectional	2021	High	29.4 (5.9)	36	Mixed or not specified	20		Placental smear microscopy	2
Mahamat, 2020 [46]	300	Cameroon	Cross Sectional	2019	High	NR (NR)	NR	Only HIV negative		98	Cord blood microscopy	1
Mama, 2022 [47]	499	Ghana	Cross Sectional	2016–19	High	26.98 (4.76)	23	Only HIV negative	91	100	Placental blood RT-PCR	1
Massamba, 2022 [48]	371	Republic of Congo	Cross Sectional	2014–15	High	NR (NR)	NR	Mixed or not specified			Placental smear microscopy	2
Mbachu, 2020 [49]	174	Nigeria	Prospective Cohort	2016–17	NR	29.1 (4.2)	NR	Only HIV positive			Placental smear microscopy	3
Mbouamboua, 2019 [50]	370	Republic of Congo	Cross Sectional	2014–15	High	25.9 (6.45)	41	Mixed or not specified			Placental blood RT-PCR	1
Megnekou, 2018 [51]	197	Cameroon	Cross Sectional	2013–15	High	26 (range = 16–39)*	36	Only HIV negative			Placental histology	2
Mikomangwa, 2020 [52]	1161	Tanzania	Cross Sectional	2018	Low	25 (range = 18–44)*	38	Only HIV negative	98		Placental histology	2
Mlugu, 2021 [53]	478	Tanzania	IPT-SP arm of RCT	2017–19	NR	26.6(7)	27	Only HIV negative			Placental histology	3
Mofon, 2022 [54]	326	Nigeria	Cross Sectional	2017–17	NR	28.6 (5.26)	29	Mixed or not specified			Placental histology	2
Mpogoro, 2014 [55]	431	Tanzania	Cross Sectional	2014–14	High	NR (NR)	53	Only HIV negative		61	Placental smear microscopy	1
Mudji, 2021 [56]	406	DRC	Prospective Cohort	2019	High	27.6 (6.9)	42	Mixed or not specified	72	80	Placental smear microscopy	4
Mwin, 2021 [57]	300	Ghana	Cross Sectional	2019	NR	26 (range = 18–48)*	29	Only HIV negative	59	93	Placental smear microscopy	3
Natama, 2023 [58]	661	Burkina Faso	Prospective Cohort	2013-2016	NR	26.4 (6.24)	34	Mixed or not specified			Placental histology	5
Nekaka, 2020 [59]	210	Uganda	Cross Sectional	2017–18	High	24.63 (6.45)	40	Mixed or not specified	58	23	Cord blood microscopy	3
Otuli Noël, 2020 [60]	1248	DRC	Cross Sectional	2018	NR	25.4 (6.24)	24	Mixed or not specified	95	95	Placental smear microscopy	3
Odongo, 2016 [76]	33	Uganda	Prospective Cohort	2014	NR	24.24 (3.5)	NR	Only HIV negative	86	50	Placental histology	4
Otuli Noël, 2020 [77]	153	DRC	Cross Sectional	2019	NR	NR (NR)	27	Only HIV negative			Placental smear microscopy	2
Owino, 2025 [61]	66	Kenya	Cross Sectional	2021	NR	23 (range = 18–37)	39	Mixed or not specified			Placental histology	3
Quakyi, 2019 [62]	935	Ghana	Cross Sectional	2013	NR	NR (NR)	NR	Only HIV Negative			Placental blood RT-PCR	2
Singh, 2014 [71]	203	India	Prospective Cohort	2014	Low	21.06 (3.03)	NR	Only HIV Negative			Placental smear microscopy	2
Solomon, 2020 [63]	230	Ethiopia	Cross Sectional	2019	NR	24.7 (5.1)	17	Mixed or not specified	93		Placental smear microscopy	3
Sylvester, 2016 [64]	206	Tanzania	Prospective cohort	2013–15	NR	NR (NR)	67	Only HIV negative	100		Placental histology	2
Tamir, 2023 [65]	460	Ethiopia	Cross Sectional	2021–22	NR	NR (NR) 25*	55	Only HIV negative	30		Placental blood RT-PCR	3
Toure, 2019 [66]	1000	Guinea	Cross Sectional	2017	NR	24.38 (6.2)	NR	Mixed or not specified			Placental smear microscopy	4
Umemmuo, 2020 [67]	426	Nigeria	Cross Sectional	2017	NR	31.9 (5.3)	54	Only HIV negative		100	Placental smear microscopy	3
Uwimana, 2023 [68]	582	Rwanda	RCT	2016–18	NR	28.8 (6.1)	27	Mixed or not specified	94		Placental blood RT-PCR	3
Vasquez, 2020 [73]	329	Colombia	Cross Sectional	2016–17	NR	23 (range = 20–28)*	39	Mixed or not specified	87		Placental blood RT-PCR	3
Weckman, 2021 [69]	421	Malawi	Prospective Cohort	2014–15	NR	21 (range = 19–25)*	66	Only HIV negative			Placental histology	5

IPTP – intermittent preventive treatment with sulfadoxine-pyrimethamine, ITN – insecticide-treated bed nets, NOS – Newcastle-Ottawa scale, NR – not reported, RT-PCR – reverse transcription polymerase chain reaction, SD – standard deviation, x̄ – mean

*Values are medians.

### Risk of bias in included studies.

The quality of the included studies varied markedly. Cross-sectional studies had total scores ranging from 1 to 5, while prospective observational studies received scores between 4 and 6. Overall, 95% (n/N = 49/50) of the studies had a high risk of bias in one of the 3 domains, while 11/34 (n/N = 32.5%) of cross-sectional studies either used convenience sampling or provided no description on how pregnant women were sampled. Conversely, ~50% had justified sample sizes and 30% relied on histological diagnosis, resulting in them having low risk of bias in these domains (Figures S1 and S2 in the **Online Supplementary Document**).

### Overall PM prevalence

**Figure 1:**
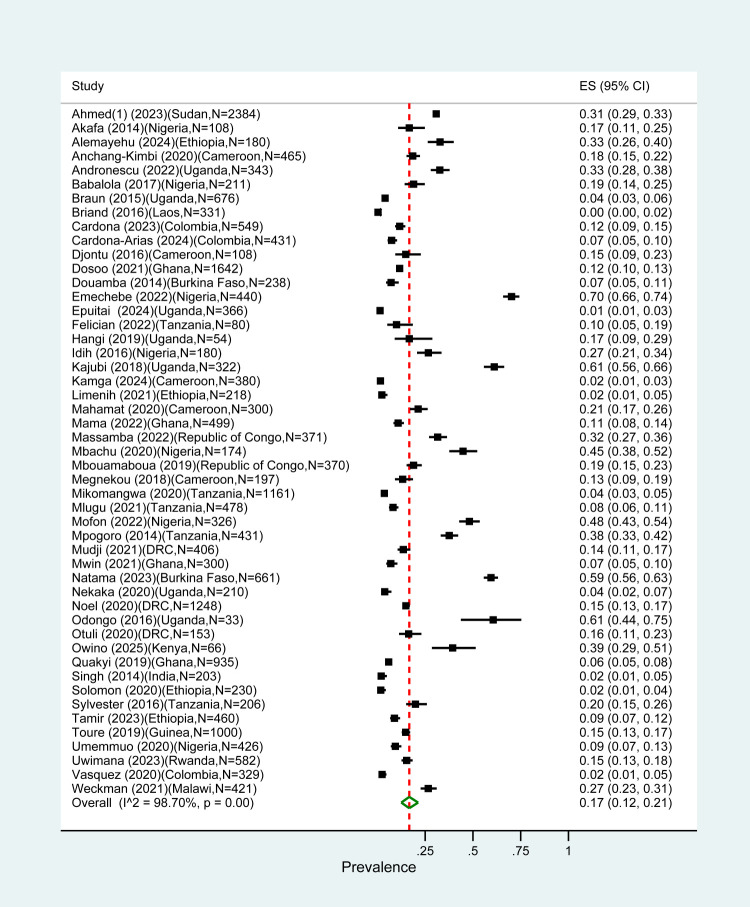
Pooled global PM prevalence.

**Figure 2:**
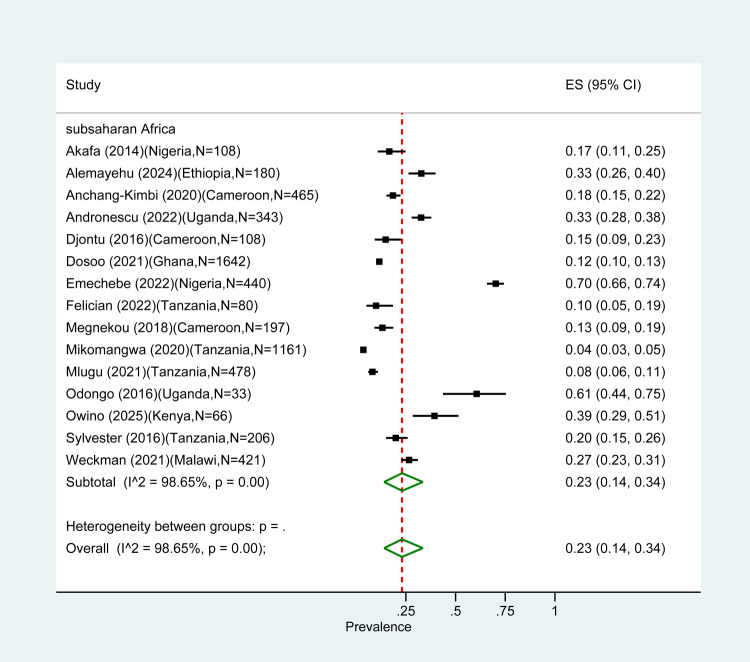
Pooled PM prevalence in sub-Saharan Africa.

**Figure 3:**
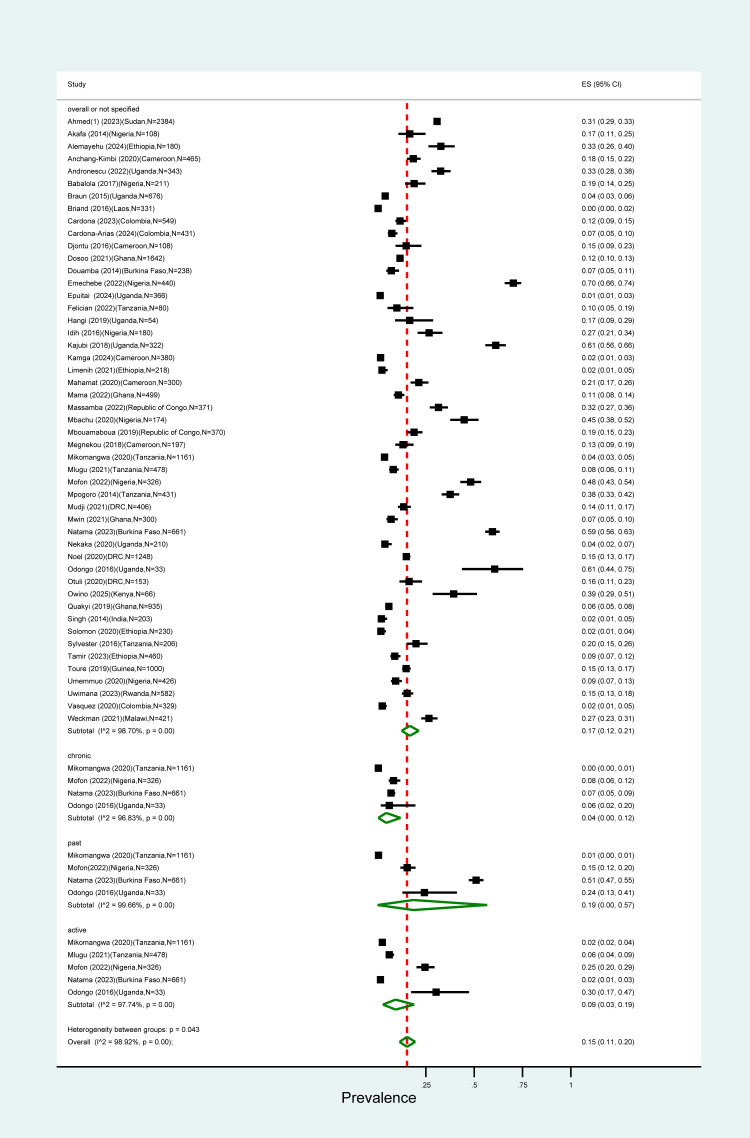
Pooled global PM prevalence by infection status.

The overall pooled prevalence for all types of PM diagnosis in pregnant women was 17% (95% confidence interval (CI) = 12–21) with an *I*^2^ value of 98.92% suggesting large heterogeneity. Of the 50 studies in our review, 15 (30%) diagnosed PM by histology. Their pooled PM prevalence was higher (23%; 95% CI = 14–34) compared to the one estimated in studies using other methods (**Figure 1**, **Figure 2**). Pooled prevalences by histology on the studies reporting by PM category were 9% (95% CI = 3–19) for acute, 4% (95% CI = 0–12) for chronic, and 19% (95% CI = 0–57) for past infections (**Figure 3**).

### Regional, subregional, and national PM prevalence

Pooled data from the studies conducted in SSA showed a higher prevalence of PM (19%; 95% CI = 14–24) compared to other regions (4%, 95% CI = 1–9) (Figure S4 in the **Online Supplementary Document**). Within SSA, West Africa had the highest prevalence, both overall (23%; 95% CI = 14–34) and based on histological methods (30%; 95% CI = 7–61), while the lowest prevalence was observed in Central Africa (overall: 16% (95% CI = 11–21); by histology: 16% (95% CI = 13–20)) (Figures S5 and S8 in the **Online Supplementary Document**). Prevalence between and within countries also varied markedly (Figure S9 in the **Online Supplementary Document**)

### Co-morbidity with HIV

One study [49] conducted in Nigeria included only HIV-positive subjects (Figures S6 and S10 in the **Online Supplementary Document**). It reported a higher PM prevalence of 45% (45%; 95% CI = 38–52) than observed in studies on HIV-negative subjects (17%; 95% CI = 10–25) and mixed or unspecified populations (0.16%; 95% CI = 0.11–0.22).

### Transmission and setting

Twenty-three studies reporting on transmission showed that high transmission areas displayed the highest overall prevalence of PM (17%; 95% CI = 11–25), while low transmission areas had lower prevalences (3%; 95% CI = 2–4) (Figure S7 in the **Online Supplementary Document**). Histological diagnosis revealed even greater disparity, with high transmission settings showing a much higher prevalence (29%; 95% CI = 10–53) compared to low transmission settings (4; 95% CI = 3–5) (Figure S11 in the **Online Supplementary Document**). A sensitivity analysis, which classified all unreported studies on transmission as either high or low, demonstrated no discernible impact on the overall findings.

### Risk factors for PM

To explore primigravida status as a risk factor, we measured a pooled OR of 1.61 (95% CI = 0.91–2.84) (Figure S13 in the **Online Supplementary Document**). We found a pooled OR of 1.26 (95% CI: 0. 74–2.15) for younger age; however, this could be attributed to the variability in age group definitions across studies (Figure S13 in the **Online Supplementary Document**). One study reported an increase in odds of PM in women not using IPTp-SP (unadjusted OR = 2.6; 95% CI = 1.2–5.4) and non-usage of INTs (unadjusted OR = 2.7; 95% CI = 1.3–5.5) [34], while those receiving >3 IPTp-SP doses were significantly less likely to have PM (OR = 0.3; 95% CI = 0.1–0.95) [55].

### Foetal outcomes associated with PM

We found no clear associations for congenital malaria, LBW, malaria in infancy, and preterm birth, as the CIs crossed the line of no effect. Notably, stillbirth was associated with PM (OR = 3.81; 95% CI = 1.22–11.94) based on a single study. For malaria in infancy (range of follow-up: 12–24 months after birth), the hazard ratio was 1.13 (95% CI = 0.66–1.93), with one study reporting an OR of 4.79 (95% CI = 2.21–10.38). The meta-analysis was confounded by variability in effect measures (hazard ratio, OR, RR) across studies and the non-rare nature of the outcomes, making the pooling of RRs unfeasible. The LBW ORs ranged [39] from 0.56 (95% CI = 0.21–1.48) to 4.67 (95% CI = 1.74–12.56), indicating substantial variability. (Figure S14 in the **Online Supplementary Document**).

### Maternal outcomes associated with PM

Only one of the 50 studies in our analysis reported on maternal anaemia [39]. It found that 12.3% of 309 pregnant women diagnosed with PM had anaemia (defined as haemoglobin levels below 11 g/dL) *vs*. 16% of pregnant women without PM.

## DISCUSSION

To our knowledge, this is the first meta-analysis to assess the burden of PM caused by *P. falciparum* and its association with adverse foetal and maternal outcomes from 2013 onwards. Our findings may help contextualise PM prevalence in the setting of recent control policies, interventions, and the emergence of drug and insecticide resistance.

The majority of the 50 studies included in our analyses was conducted in SSA countries, and 30% relied on histological diagnosis, which is the gold standard for detecting PM. We estimated the overall global prevalence of PM attributed to *P. falciparum* with histological diagnosis to be approximately one third of all pregnancies in malaria-endemic areas. The pooled prevalence of PM in SSA measured by all diagnostic methods was approximately one in five women, compared to one in twenty-five in Asia and Latin America. High transmission settings in SSA, particularly in Western Africa, show significantly higher prevalence rates.

In studies reporting on acute, chronic, and past infections by histology, we found that approximately 50% of the pooled prevalence was due to past placental infections. This indicates that while active infections at the time of delivery (acute and/or chronic) are low, significant prior exposure to *P. falciparum* occurred during early phases of pregnancy. Despite these infections having been resolved at the time of delivery, they may nevertheless result in placental alterations and poor clinical outcomes in pregnant women and their infants. Consequently, reliance on limited diagnostic methods may lead to a large underestimation of PM cases, potentially missing more than 50% of the true burden. Histology, in turn, may capture both current and past PM exposure.

Residual heterogeneity in this meta-analysis on PM prevalence may be attributed to diverse factors, including socioeconomic disparities [78], sociocultural aspects [79], and genetic factors such as co-occurrence of glucose-6-phosphate dehydrogenase deficiency and sickle cell trait [80,81], all of which modulate malaria susceptibility and prevalence. Additionally, environmental conditions and variability in adherence to IPTp protocols may also contribute to the variability. This heterogeneity is consistent with findings showing that malaria transmission may vary substantially at sub-national levels [82–85]. However, we note that approximately half of the included studies did not describe transmission setting, meaning that some of the variation in PM prevalence by country may stem from this unmeasured variable. Nonetheless, a substantial proportion of variability remains unexplained by the model.

Our meta-analysis identified an inconclusive association between primigravity and PM, contrasting with existing literature [86], which may be due to our small sample size. Younger maternal age could potentially correlate with increased risk of PM; however, comparability is hampered by high variability in age definitions among studies. The ORs and CIs for congenital malaria, LBW, malaria in infancy, and preterm birth showed substantial variability and imprecision in the estimates. Notably, malaria in infancy and stillbirth were associated with higher odds in a single study. While the point estimates suggest an increased risk, the wide confidence intervals indicate substantial uncertainty consistent with prior evidence from a systematic review including studies prior to 2013, suggesting that PM caused by *P. falciparum* increased the risk of stillbirth [87] and clinically defined malaria in young children [88].Adverse foetal outcomes may be multifactorial, with PM possibly being a critical but not sole determinant. Due to insufficient sample size of studies exploring these associations and consequent imprecision, we are not able to ascertain whether this holds true. Destruction of maternal red blood cell, prominent in cases of peripheral malaria, may also contribute to these adverse outcomes, here again, suboptimal sample size precludes definitive conclusions.

Our findings entail certain public health implications. Surveillance systems that rely on non-histological diagnoses likely underestimate the true burden of PM. The current PM prevention landscape, including ITNs and IPTp-SP, suffers from inconsistent reporting and adherence, and appears insufficient, underscoring the need to strengthen preventive measures. Specific PM vaccines, such as VAR2CSA-derived candidates, may eventually offer additional protection for primigravid in high-transmission areas, although clinical trials are still at an early stage [21,22]. Evidence from a single study suggests that HIV-positive pregnant women could have a higher prevalence of PM, which is biologically plausible given immune suppression, but data are limited and estimates are imprecise [49]. Our findings also underscore the need to prioritise high-risk groups, including HIV-positive and primigravid women, in future interventions, while erring on the side of caution in interpreting evidence.

Evidence suggests that the burden of PM due to *P. falciparum* outside SSA is low. However, in low-transmission areas, where conditions favour the re-emergence of *P. falciparum*, women may face a higher risk of adverse maternal and foetal outcomes due to lack of immunity, with climate change potentially turning these areas into future hotspots. The heterogeneity observed in our meta-analyses highlights the need for region-specific interventions to improve PM prevention and management

### Strengths and limitations

Our systematic review on PM has several strengths. By focussing on studies conducted from 2013 onwards, we provide a current assessment of pooled PM prevalence following important changes in malaria control and elimination strategies in many endemic countries. Our review did not exclude studies based on languages, enhancing its global relevance. Importantly, about one third of the included studies employed histological methods for PM diagnosis, providing greater precision than routine diagnostics, yet at the trade-off in terms of practicality for surveillance due to its labour and resource requirement.

However, despite our attempts to explore potential sources of heterogeneity by diagnostic method, HIV status, transmission setting, and geographical regions, several limitations should be acknowledged. Missing sociodemographic information and the lack of standardisation of reporting precluded us from contextualising many studies. As a result, we could not consistently ascertain the proportion of pregnant women who utilised IPTp and ITNs, nor confirm the number of IPTp doses received. This limitation prevented us from conducting sub-analyses to explore heterogeneity by these factors. Furthermore, seasonality was generally unreported in these studies, and while pregnancies often span multiple seasons, potentially mitigating seasonal effects, its influence cannot be fully excluded. Many studies only indicated that they occurred in endemic settings without detailing transmission intensity. Nevertheless, our sensitivity analysis, which categorised all unreported studies on transmission as either high or low, demonstrated no significant impact on the overall findings, reinforcing the robustness of our pooled prevalence estimates.

Inconsistent and non-standardised sociodemographic and clinical data, along with variability in the collection of risk factor information, limited our ability to conduct additional subgroup analyses and constrained multivariate meta-regression performance. Clinical variables such as gravidity, parity, and co-infections were not consistently reported. Only one single study specially examined HIV+ women in relation to PM and adverse pregnancy outcomes [49], raising concerns about the precision of subgroup estimates and limiting our ability to fully identify which populations of pregnant women are at highest risk.

Thus, the residual heterogeneity likely reflects the inclusion of diverse populations, geographic regions, and varying demographic, nutritional, and clinical profiles. While this real-world diversity enhances generalisability and strengthens external validity, the extremely high heterogeneity also limits the precision and interpretability of pooled prevalence estimates. As a corollary, the pooled estimates should be interpreted as indicative rather than precise point estimates. Likewise, lack of data prevented us from conducting a meta-analysis on the association between maternal and certain foetal outcomes and PM.

We also note that we excluded one study [89] overlapping the pre- and post-2013 periods, because data were not disaggregated by year. Whilst this may have led to the omission of some relevant information, it is unlikely to have affected the pooled prevalence estimates. Moreover, the quality of the studies varied substantially, with approximately half justifying their sample sizes and one third of studies either not reporting on their sampling methods or relying on convenience sampling at a single site. This may have introduced a selection bias, which in turn may have either inflated or underestimated prevalence estimates. While cross-sectional designs are amenable to determining the burden of PM, they limit causal inference and the ability fully account for confounding factors when exploring associations with adverse maternal or foetal outcomes. These methodological limitations, along with variability in study quality and reporting, may have further contributed to the high heterogeneity observed across studies. Additionally, the small number of studies in our review precluded formal assessment of publication bias, leaving open the possibility that non-published studies could have affected the pooled estimates.

Furthermore, it is important to consider the external validity of our findings, as only women who accessed antenatal care were included in the studies. Consequently, women without access to antenatal care, who are likely at highest risk for PM due to reduced access to preventive interventions such as IPTp-SP and ITN, as well as increased vulnerability form malnutrition, untreated co-infections, were not captured, potentially introducing selection bias and underestimating the true burden in the broader population. Finally, most included studies (n/N = 45/50) were conducted in SSA, with only five originating from other regions, thereby limiting the global representativeness of our findings.

Future research should also prioritise the standardisation of methodologies to investigate the relationship between PM and specific maternal and foetal outcomes. Simultaneously, more consistent reporting of sociodemographic data will elucidate risk factors and help inform targeted prevention strategies.

## CONCLUSIONS

Our meta-analysis suggests that about one out of six pregnant women in all disease-endemic settings experiences PM caused by *P. falciparum*. In SSA, this figure is one out of five. When looking exclusively at studies using histological methods, widely regarded as the gold standard for PM diagnosis, almost one out of four women in SSA experiences PM. As surveillance systems frequently depend on non-histological diagnostic methods, they may underestimate the true burden of PM, particularly past or low-density infections, thus highlighting the need for more feasible and more accurate diagnostic paradigms to capture the true burden of PM, while considering practical constraints in routine surveillance.

While primigravidae had a 61% higher odds of PM compared to multigravidae, this result should be interpreted with caution due to the imprecision of the pooled estimates in our meta-analysis. The scarcity of studies including only HIV+ women limits conclusions for this group. The point estimates for congenital malaria, LBW, and malaria in infancy, indicate a potential increase in odds, yet this finding, too, is limited by the imprecision of our pooled results. Data suggests that PM may increase the odds of stillbirth, but more investigations will be required to confirm this finding. Further research is needed to fully understand the link between PM and HIV as well as any link between PM and foetal and maternal outcomes to optimally support current and future public health interventions, especially in high transmission areas.

## Additional material


Online Supplementary Document
