# Supplementary material for: Placental malaria: a systematic review and meta-analysis of global burden, risk factors, and maternal and foetal outcomes
Source: J Glob Health. 2025 Nov 28;15:04355. doi: 10.7189/jogh.15.04355 (PMC12659800; doi:10.7189/jogh.15.04355)
Supplement: Online Supplementary Document [file jogh-15-04355-s001.pdf]

Supplement to: Menon S, D'Alessio F, Chaudhuri N, Onwuchekwa C, Kaur M, Nkumama I, Olesen OF. Placental malaria: a systematic review and meta-analysis of global burden, risk factors, and maternal and foetal outcomes. J Glob Health. 2025;15:04355.

By **Sonia Menon*, Flavia D'Alessio*, Nita Chaudhuri, Chukwuemeka Onwuchekwa, Mandeep Kaur, Irene Nkumama, Ole F. Olesen**

Contents

Search strategy 3
Table of excluded full text screened studies 11
Risk of bias assessment 18
Summary plot: all outcomes 19
Additional supplementary figures 20
Subgroup analysis by histology 25
Risk factors for PM 29
Foetal outcomes associated with PM 31
.... 31

Search strategy

We searched the following databases: Medline Ovid *23/03/2025), Embase (23/03/2025), Scopus (24/03/2025).

MEDLINE Ovid (2013 to March 2025 (2,161 hits)

#10 OR #11 OR #12 OR #13 ((("malaria in pregnancy"[Title/Abstract] OR "placental malaria"[Title/Abstract] OR "antenatal malaria"[Title/Abstract] OR ("pregnancy"[Title/Abstract] AND "Malaria"[Title/Abstract]) OR ("obstetric"[Title/Abstract] AND "Malaria"[Title/Abstract]) OR (("obstetric"[All Fields] OR "obstetrically"[All Fields] OR "obstetrics"[MeSH Terms] OR "obstetrics"[All Fields] OR "obstetrical"[All Fields]) AND "Malaria"[Title/Abstract]) OR ("Plasmodium"[Title/Abstract] AND "pregnancy"[Title/Abstract]) OR (("malaria, falciparum"[MeSH Terms] OR "malaria, falciparum"[MeSH Terms] OR "plasmodium ovale"[MeSH Terms] OR "malaria, vivax"[MeSH Terms]) AND ("pregnant women"[MeSH Terms] OR "pregnant women"[MeSH Terms]))) AND ("prevalence"[Title/Abstract] OR "incidence"[Title/Abstract] OR "burden"[Title/Abstract] OR "epidemiology"[Title/Abstract] OR "risk"[Title/Abstract] OR "rate"[Title/Abstract] OR "seroprevalence"[Title/Abstract] OR "prevalence"[MeSH Terms] OR "incidence"[MeSH Terms] OR "cost of illness"[MeSH Terms] OR "epidemiology"[MeSH Terms])) OR (("malaria in pregnancy"[Title/Abstract] OR "placental malaria"[Title/Abstract] OR "antenatal malaria"[Title/Abstract] OR ("pregnancy"[Title/Abstract] AND "Malaria"[Title/Abstract]) OR ("obstetric"[Title/Abstract] AND "Malaria"[Title/Abstract]) OR (("obstetric"[All Fields] OR "obstetrically"[All Fields] OR "obstetrics"[MeSH Terms] OR "obstetrics"[All Fields] OR "obstetrical"[All Fields]) AND "Malaria"[Title/Abstract]) OR ("Plasmodium"[Title/Abstract] AND "pregnancy"[Title/Abstract]) OR (("malaria, falciparum"[MeSH Terms] OR "malaria, falciparum"[MeSH Terms] OR "plasmodium ovale"[MeSH Terms] OR "malaria, vivax"[MeSH Terms]) AND ("pregnant women"[MeSH Terms] OR "pregnant women"[MeSH Terms]))) AND ("anemia, iron deficiency"[MeSH Terms] OR ("anaemia"[Title/Abstract] OR "anemia"[Title/Abstract]) OR "cerebral malaria"[Title/Abstract] OR "malaria, cerebral"[MeSH Terms] OR "malaria, cerebral"[MeSH Terms] OR "maternal mortality"[MeSH Terms] OR "maternal death"[Title/Abstract] OR "mortality"[Title/Abstract] OR "severe malaria"[Title/Abstract] OR "hyperparasitaemia"[Title/Abstract] OR "hyperparasitemia"[Title/Abstract] OR "maternal complication"[Title/Abstract] OR "pregnancy complications"[MeSH Terms])) OR (("malaria in pregnancy"[Title/Abstract] OR "placental malaria"[Title/Abstract] OR "antenatal malaria"[Title/Abstract] OR ("pregnancy"[Title/Abstract] AND "Malaria"[Title/Abstract]) OR ("obstetric"[Title/Abstract] AND "Malaria"[Title/Abstract]) OR (("obstetric"[All Fields] OR "obstetrically"[All Fields] OR "obstetrics"[MeSH Terms] OR "obstetrics"[All Fields] OR "obstetrical"[All Fields]) AND "Malaria"[Title/Abstract]) OR ("Plasmodium"[Title/Abstract] AND "pregnancy"[Title/Abstract]) OR (("malaria, falciparum"[MeSH Terms] OR "malaria, falciparum"[MeSH Terms] OR "plasmodium ovale"[MeSH Terms] OR "malaria, vivax"[MeSH Terms]) AND ("pregnant women"[MeSH Terms] OR "pregnant women"[MeSH Terms]))) AND ("abortion, spontaneous"[MeSH Terms] OR "abortion, spontaneous"[MeSH Terms] OR "stillbirth"[MeSH Terms] OR "premature birth"[MeSH

Terms] OR "premature birth"[MeSH Terms] OR "fetal growth retardation"[MeSH Terms] OR ((("retard"[All Fields] OR "retardance"[All Fields] OR "retardances"[All Fields] OR "retardation"[All Fields] OR "retardations"[All Fields] OR "retarded"[All Fields] OR "retarder"[All Fields] OR "retarders"[All Fields] OR "retarding"[All Fields] OR "retards"[All Fields]) AND ("intrauterin"[All Fields] OR "intrauterine"[All Fields])) AND "growth"[MeSH Terms]) OR "infant, low birth weight"[MeSH Terms] OR "infant mortality"[MeSH Terms] OR "infant mortality"[MeSH Terms] OR ("abortion"[Title/Abstract] OR "miscarriage"[Title/Abstract] OR "stillbirth"[Title/Abstract] OR "premature birth"[Title/Abstract] OR "preterm"[Title/Abstract] OR "intrauterine growth retardation"[Title/Abstract] OR "IUGR"[Title/Abstract] OR "congenital malaria"[Title/Abstract] OR "mortality"[Title/Abstract] OR "death"[Title/Abstract] OR "complication"[Title/Abstract] OR ("sequealae"[All Fields] AND "OR"[Title/Abstract])) OR ("anemia"[Title/Abstract] OR "anaemia"[Title/Abstract]))) OR (("malaria in pregnancy"[Title/Abstract] OR "placental malaria"[Title/Abstract] OR "antenatal malaria"[Title/Abstract] OR ("pregnancy"[Title/Abstract] AND "Malaria"[Title/Abstract]) OR ("obstetric"[Title/Abstract] AND "Malaria"[Title/Abstract]) OR (("obstetric"[All Fields] OR "obstetrically"[All Fields] OR "obstetrics"[MeSH Terms] OR "obstetrics"[All Fields] OR "obstetrical"[All Fields]) AND "Malaria"[Title/Abstract]) OR ("Plasmodium"[Title/Abstract] AND "pregnancy"[Title/Abstract]) OR (("malaria, falciparum"[MeSH Terms] OR "malaria, falciparum"[MeSH Terms] OR "plasmodium ovale"[MeSH Terms] OR "malaria, vivax"[MeSH Terms]) AND ("pregnant women"[MeSH Terms] OR "pregnant women"[MeSH Terms]))) AND ("risk factors"[MeSH Terms] OR ("risk factor"[Title/Abstract] OR "risk group"[Title/Abstract]) OR ("parity"[Title/Abstract] OR "gravidity"[Title/Abstract] OR "age"[Title/Abstract] OR "transmission intensity"[Title/Abstract] OR "endemicity"[Title/Abstract] OR "season"[Title/Abstract] OR "prophylaxis"[Title/Abstract] OR "bednet"[Title/Abstract] OR "socioeconomic"[Title/Abstract]) OR "parity"[MeSH Terms] OR "gravidity"[MeSH Terms] OR "insecticide treated bednets"[MeSH Terms] OR "endemic diseases"[MeSH Terms] OR "social class"[MeSH Terms] OR ("ethnology"[MeSH Terms] OR "ethnicity"

#10 OR #11 OR #12 OR #13 (("malaria in pregnancy"[Title/Abstract] OR "placental malaria"[Title/Abstract] OR "antenatal malaria"[Title/Abstract] OR ("pregnancy"[Title/Abstract] AND "Malaria"[Title/Abstract]) OR ("obstetric"[Title/Abstract] AND "Malaria"[Title/Abstract]) OR (("obstetric"[All Fields] OR "obstetrically"[All Fields] OR "obstetrics"[MeSH Terms] OR "obstetrics"[All Fields] OR "obstetrical"[All Fields]) AND "Malaria"[Title/Abstract]) OR ("Plasmodium"[Title/Abstract] AND "pregnancy"[Title/Abstract]) OR (("malaria, falciparum"[MeSH Terms] OR "malaria, falciparum"[MeSH Terms] OR "plasmodium ovale"[MeSH Terms] OR "malaria, vivax"[MeSH Terms]) AND ("pregnant women"[MeSH Terms] OR "pregnant women"[MeSH Terms]))) AND ("prevalence"[Title/Abstract] OR "incidence"[Title/Abstract] OR "burden"[Title/Abstract] OR "epidemiology"[Title/Abstract] OR "risk"[Title/Abstract] OR "rate"[Title/Abstract] OR "seroprevalence"[Title/Abstract] OR "prevalence"[MeSH Terms] OR "incidence"[MeSH Terms] OR "cost of illness"[MeSH Terms] OR "epidemiology"[MeSH Terms])) OR (("malaria in pregnancy"[Title/Abstract] OR "placental malaria"[Title/Abstract] OR "antenatal malaria"[Title/Abstract] OR ("pregnancy"[Title/Abstract] AND "Malaria"[Title/Abstract]) OR ("obstetric"[Title/Abstract] AND "Malaria"[Title/Abstract]) OR (("obstetric"[All Fields] OR "obstetrically"[All Fields]

OR "obstetrics"[MeSH Terms] OR "obstetrics"[All Fields] OR "obstetrical"[All Fields]) AND "Malaria"[Title/Abstract]) OR ("Plasmodium"[Title/Abstract] AND "pregnancy"[Title/Abstract]) OR (("malaria, falciparum"[MeSH Terms] OR "malaria, falciparum"[MeSH Terms] OR "plasmodium ovale"[MeSH Terms] OR "malaria, vivax"[MeSH Terms]) AND ("pregnant women"[MeSH Terms] OR "pregnant women"[MeSH Terms]))) AND ("anemia, iron deficiency"[MeSH Terms] OR ("anaemia"[Title/Abstract] OR "anemia"[Title/Abstract]) OR "cerebral malaria"[Title/Abstract] OR "malaria, cerebral"[MeSH Terms] OR "malaria, cerebral"[MeSH Terms] OR "maternal mortality"[MeSH Terms] OR "maternal death"[Title/Abstract] OR "mortality"[Title/Abstract] OR "severe malaria"[Title/Abstract] OR "hyperparasitaemia"[Title/Abstract] OR "hyperparasitemia"[Title/Abstract] OR "maternal complication"[Title/Abstract] OR "pregnancy complications"[MeSH Terms])) OR (("malaria in pregnancy"[Title/Abstract] OR "placental malaria"[Title/Abstract] OR "antenatal malaria"[Title/Abstract] OR ("pregnancy"[Title/Abstract] AND "Malaria"[Title/Abstract]) OR ("obstetric"[Title/Abstract] AND "Malaria"[Title/Abstract]) OR (("obstetric"[All Fields] OR "obstetrically"[All Fields] OR "obstetrics"[MeSH Terms] OR "obstetrics"[All Fields] OR "obstetrical"[All Fields]) AND "Malaria"[Title/Abstract]) OR ("Plasmodium"[Title/Abstract] AND "pregnancy"[Title/Abstract]) OR (("malaria, falciparum"[MeSH Terms] OR "malaria, falciparum"[MeSH Terms] OR "plasmodium ovale"[MeSH Terms] OR "malaria, vivax"[MeSH Terms]) AND ("pregnant women"[MeSH Terms] OR "pregnant women"[MeSH Terms]))) AND ("abortion, spontaneous"[MeSH Terms] OR "abortion, spontaneous"[MeSH Terms] OR "stillbirth"[MeSH Terms] OR "premature birth"[MeSH Terms] OR "premature birth"[MeSH Terms] OR "fetal growth retardation"[MeSH Terms] OR ((("retard"[All Fields] OR "retardance"[All Fields] OR "retardances"[All Fields] OR "retardation"[All Fields] OR "retardations"[All Fields] OR "retarded"[All Fields] OR "retarder"[All Fields] OR "retarders"[All Fields] OR "retarding"[All Fields] OR "retards"[All Fields]) AND ("intrauterin"[All Fields] OR "intrauterine"[All Fields])) AND "growth"[MeSH Terms]) OR "infant, low birth weight"[MeSH Terms] OR "infant mortality"[MeSH Terms] OR "infant mortality"[MeSH Terms] OR ("abortion"[Title/Abstract] OR "miscarriage"[Title/Abstract] OR "stillbirth"[Title/Abstract] OR "premature birth"[Title/Abstract] OR "preterm"[Title/Abstract] OR "intrauterine growth retardation"[Title/Abstract] OR "IUGR"[Title/Abstract] OR "congenital malaria"[Title/Abstract] OR "mortality"[Title/Abstract] OR "death"[Title/Abstract] OR "complication"[Title/Abstract] OR ("sequealae"[All Fields] AND "OR"[Title/Abstract])) OR ("anemia"[Title/Abstract] OR "anaemia"[Title/Abstract]))) OR (("malaria in pregnancy"[Title/Abstract] OR "placental malaria"[Title/Abstract] OR "antenatal malaria"[Title/Abstract] OR ("pregnancy"[Title/Abstract] AND "Malaria"[Title/Abstract]) OR ("obstetric"[Title/Abstract] AND "Malaria"[Title/Abstract]) OR (("obstetric"[All Fields] OR "obstetrically"[All Fields] OR "obstetrics"[MeSH Terms] OR "obstetrics"[All Fields] OR "obstetrical"[All Fields]) AND "Malaria"[Title/Abstract]) OR ("Plasmodium"[Title/Abstract] AND "pregnancy"[Title/Abstract]) OR (("malaria, falciparum"[MeSH Terms] OR "malaria, falciparum"[MeSH Terms] OR "plasmodium ovale"[MeSH Terms] OR "malaria, vivax"[MeSH Terms]) AND ("pregnant women"[MeSH Terms] OR "pregnant women"[MeSH Terms]))) AND ("risk factors"[MeSH Terms] OR ("risk factor"[Title/Abstract] OR "risk group"[Title/Abstract]) OR ("parity"[Title/Abstract] OR "gravidity"[Title/Abstract] OR "age"[Title/Abstract] OR "transmission

intensity"[Title/Abstract] OR "endemicity"[Title/Abstract] OR "season"[Title/Abstract] OR "prophylaxis"[Title/Abstract] OR "bednet"[Title/Abstract] OR "socioeconomic"[Title/Abstract]) OR "parity"[MeSH Terms] OR "gravidity"[MeSH Terms] OR "insecticide treated bednets"[MeSH Terms] OR "endemic diseases"[MeSH Terms] OR "social class"[MeSH Terms] OR ("ethnology"[MeSH Terms] OR "ethnicity"[MeSH Terms]))): 3,910 hits

#5 AND #9 ("malaria in pregnancy"[Title/Abstract] OR "placental malaria"[Title/Abstract] OR "antenatal malaria"[Title/Abstract] OR ("pregnancy"[Title/Abstract] AND "Malaria"[Title/Abstract]) OR ("obstetric"[Title/Abstract] AND "Malaria"[Title/Abstract]) OR (("obstetric"[All Fields] OR "obstetrically"[All Fields] OR "obstetrics"[MeSH Terms] OR "obstetrics"[All Fields] OR "obstetrical"[All Fields]) AND "Malaria"[Title/Abstract]) OR ("Plasmodium"[Title/Abstract] AND "pregnancy"[Title/Abstract]) OR (("malaria, falciparum"[MeSH Terms] OR "malaria, falciparum"[MeSH Terms] OR "plasmodium ovale"[MeSH Terms] OR "malaria, vivax"[MeSH Terms]) AND ("pregnant women"[MeSH Terms] OR "pregnant women"[MeSH Terms]))) AND ("risk factors"[MeSH Terms] OR ("risk factor"[Title/Abstract] OR "risk group"[Title/Abstract]) OR ("parity"[Title/Abstract] OR "gravidity"[Title/Abstract] OR "age"[Title/Abstract] OR "transmission intensity"[Title/Abstract] OR "endemicity"[Title/Abstract] OR "season"[Title/Abstract] OR "prophylaxis"[Title/Abstract] OR "bednet"[Title/Abstract] OR "socioeconomic"[Title/Abstract]) OR "parity"[MeSH Terms] OR "gravidity"[MeSH Terms] OR "insecticide treated bednets"[MeSH Terms] OR "endemic diseases"[MeSH Terms] OR "social class"[MeSH Terms] OR ("ethnology"[MeSH Terms] OR "ethnicity"[MeSH Terms])): 1,978 hits

#5 AND #8 ("malaria in pregnancy"[Title/Abstract] OR "placental malaria"[Title/Abstract] OR "antenatal malaria"[Title/Abstract] OR ("pregnancy"[Title/Abstract] AND "Malaria"[Title/Abstract]) OR ("obstetric"[Title/Abstract] AND "Malaria"[Title/Abstract]) OR (("obstetric"[All Fields] OR "obstetrically"[All Fields] OR "obstetrics"[MeSH Terms] OR "obstetrics"[All Fields] OR "obstetrical"[All Fields]) AND "Malaria"[Title/Abstract]) OR ("Plasmodium"[Title/Abstract] AND "pregnancy"[Title/Abstract]) OR (("malaria, falciparum"[MeSH Terms] OR "malaria, falciparum"[MeSH Terms] OR "plasmodium ovale"[MeSH Terms] OR "malaria, vivax"[MeSH Terms]) AND ("pregnant women"[MeSH Terms] OR "pregnant women"[MeSH Terms]))) AND ("abortion, spontaneous"[MeSH Terms] OR "abortion, spontaneous"[MeSH Terms] OR "stillbirth"[MeSH Terms] OR "premature birth"[MeSH Terms] OR "premature birth"[MeSH Terms] OR "fetal growth retardation"[MeSH Terms] OR ((("retard"[All Fields] OR "retardance"[All Fields] OR "retardances"[All Fields] OR "retardation"[All Fields] OR "retardations"[All Fields] OR "retarded"[All Fields] OR "retarder"[All Fields] OR "retarders"[All Fields] OR "retarding"[All Fields] OR "retards"[All Fields]) AND ("intrauterin"[All Fields] OR "intrauterine"[All Fields])) AND "growth"[MeSH Terms]) OR "infant, low birth weight"[MeSH Terms] OR "infant mortality"[MeSH Terms] OR "infant mortality"[MeSH Terms] OR ("abortion"[Title/Abstract] OR "miscarriage"[Title/Abstract] OR "stillbirth"[Title/Abstract] OR "premature birth"[Title/Abstract] OR "preterm"[Title/Abstract] OR "intrauterine growth retardation"[Title/Abstract] OR "IUGR"[Title/Abstract] OR "congenital malaria"[Title/Abstract] OR "mortality"[Title/Abstract] OR "death"[Title/Abstract] OR "complication"[Title/Abstract] OR ("sequealae"[All Fields] AND

"OR"[Title/Abstract])) OR ("anemia"[Title/Abstract] OR "anaemia"[Title/Abstract])); hits 2,066

#5 AND #7 ("malaria in pregnancy"[Title/Abstract] OR "placental malaria"[Title/Abstract] OR "antenatal malaria"[Title/Abstract] OR ("pregnancy"[Title/Abstract] AND "Malaria"[Title/Abstract]) OR ("obstetric"[Title/Abstract] AND "Malaria"[Title/Abstract]) OR (("obstetric"[All Fields] OR "obstetrically"[All Fields] OR "obstetrics"[MeSH Terms] OR "obstetrics"[All Fields] OR "obstetrical"[All Fields]) AND "Malaria"[Title/Abstract]) OR ("Plasmodium"[Title/Abstract] AND "pregnancy"[Title/Abstract]) OR (("malaria, falciparum"[MeSH Terms] OR "malaria, falciparum"[MeSH Terms] OR "plasmodium ovale"[MeSH Terms] OR "malaria, vivax"[MeSH Terms]) AND ("pregnant women"[MeSH Terms] OR "pregnant women"[MeSH Terms]))) AND ("anemia, iron deficiency"[MeSH Terms] OR ("anaemia"[Title/Abstract] OR "anemia"[Title/Abstract]) OR "cerebral malaria"[Title/Abstract] OR "malaria, cerebral"[MeSH Terms] OR "malaria, cerebral"[MeSH Terms] OR "maternal mortality"[MeSH Terms] OR "maternal death"[Title/Abstract] OR "mortality"[Title/Abstract] OR "severe malaria"[Title/Abstract] OR "hyperparasitaemia"[Title/Abstract] OR "hyperparasitemia"[Title/Abstract] OR "maternal complication"[Title/Abstract] OR "pregnancy complications"[MeSH Terms]). Hits: 3,208

#5 AND #6 ("malaria in pregnancy"[Title/Abstract] OR "placental malaria"[Title/Abstract] OR "antenatal malaria"[Title/Abstract] OR ("pregnancy"[Title/Abstract] AND "Malaria"[Title/Abstract]) OR ("obstetric"[Title/Abstract] AND "Malaria"[Title/Abstract]) OR (("obstetric"[All Fields] OR "obstetrically"[All Fields] OR "obstetrics"[MeSH Terms] OR "obstetrics"[All Fields] OR "obstetrical"[All Fields]) AND "Malaria"[Title/Abstract]) OR ("Plasmodium"[Title/Abstract] AND "pregnancy"[Title/Abstract]) OR (("malaria, falciparum"[MeSH Terms] OR "malaria, falciparum"[MeSH Terms] OR "plasmodium ovale"[MeSH Terms] OR "malaria, vivax"[MeSH Terms]) AND ("pregnant women"[MeSH Terms] OR "pregnant women"[MeSH Terms]))) AND ("prevalence"[Title/Abstract] OR "incidence"[Title/Abstract] OR "burden"[Title/Abstract] OR "epidemiology"[Title/Abstract] OR "risk"[Title/Abstract] OR "rate"[Title/Abstract] OR "seroprevalence"[Title/Abstract] OR "prevalence"[MeSH Terms] OR "incidence"[MeSH Terms] OR "cost of illness"[MeSH Terms] OR "epidemiology"[MeSH Terms]). Hits: 2.449

Risk factors: "risk factors"[MeSH Terms] OR "risk factor"[Title/Abstract] OR "risk group"[Title/Abstract] OR "parity"[Title/Abstract] OR "gravidity"[Title/Abstract] OR "age"[Title/Abstract] OR "transmission intensity"[Title/Abstract] OR "endemicity"[Title/Abstract] OR "season"[Title/Abstract] OR "prophylaxis"[Title/Abstract] OR "bednet"[Title/Abstract] OR "socioeconomic"[Title/Abstract] OR "parity"[MeSH Terms] OR "gravidity"[MeSH Terms] OR "insecticide treated bednets"[MeSH Terms] OR "endemic diseases"[MeSH Terms] OR "social class"[MeSH Terms] OR "ethnology"[MeSH Terms] OR "ethnicity"[MeSH Terms]

Fetal and infant complications: "abortion, spontaneous"[MeSH Terms] OR "abortion, spontaneous"[MeSH Terms] OR "stillbirth"[MeSH Terms] OR "premature birth"[MeSH Terms] OR "premature birth"[MeSH Terms] OR "fetal growth retardation"[MeSH Terms] OR ((("retard"[All Fields] OR "retardance"[All Fields] OR "retardances"[All Fields] OR "retardation"[All Fields] OR "retardations"[All Fields] OR "retarded"[All Fields] OR "retarder"[All Fields] OR "retarders"[All Fields] OR "retarding"[All Fields] OR "retards"[All

Fields]) AND ("intrauterin"[All Fields] OR "intrauterine"[All Fields])) AND "growth"[MeSH Terms]) OR "infant, low birth weight"[MeSH Terms] OR "infant mortality"[MeSH Terms] OR "infant mortality"[MeSH Terms] OR ("abortion"[Title/Abstract] OR "miscarriage"[Title/Abstract] OR "stillbirth"[Title/Abstract] OR "premature birth"[Title/Abstract] OR "preterm"[Title/Abstract] OR "intrauterine growth retardation"[Title/Abstract] OR "IUGR"[Title/Abstract] OR "congenital malaria"[Title/Abstract] OR "mortality"[Title/Abstract] OR "death"[Title/Abstract] OR "complication"[Title/Abstract] OR ("sequealae"[All Fields] AND "OR"[Title/Abstract])) OR ("anemia"[Title/Abstract] OR "anaemia"[Title/Abstract])

Maternal complications

"anemia, iron deficiency"[MeSH Terms] OR "anaemia"[Title/Abstract] OR "anemia"[Title/Abstract] OR "cerebral malaria"[Title/Abstract] OR "malaria, cerebral"[MeSH Terms] OR "malaria, cerebral"[MeSH Terms] OR "maternal mortality"[MeSH Terms] OR "maternal death"[Title/Abstract] OR "mortality"[Title/Abstract] OR "severe malaria"[Title/Abstract] OR "hyperparasitaemia"[Title/Abstract] OR "hyperparasitemia"[Title/Abstract] OR "maternal complication"[Title/Abstract] OR "pregnancy complications"[MeSH Terms]

Prevalence, incidence terms: "prevalence"[Title/Abstract] OR “incidence"[Title/Abstract] OR "burden"[Title/Abstract] OR "epidemiology"[Title/Abstract] OR "risk"[Title/Abstract] OR "rate"[Title/Abstract] OR "seroprevalence"[Title/Abstract] OR "prevalence"[MeSH Terms] OR "incidence"[MeSH Terms] OR "cost of illness"[MeSH Terms] OR "epidemiology"[MeSH Terms]

#1 OR #4 "malaria in pregnancy"[Title/Abstract] OR "placental malaria"[Title/Abstract] OR "antenatal malaria"[Title/Abstract] OR ("pregnancy"[Title/Abstract] AND "Malaria"[Title/Abstract]) OR ("obstetric"[Title/Abstract] AND "Malaria"[Title/Abstract]) OR (("obstetric"[All Fields] OR "obstetrically"[All Fields] OR "obstetrics"[MeSH Terms] OR "obstetrics"[All Fields] OR "obstetrical"[All Fields]) AND "Malaria"[Title/Abstract]) OR ("Plasmodium"[Title/Abstract] AND "pregnancy"[Title/Abstract]) OR (("malaria, falciparum"[MeSH Terms] OR "malaria, falciparum"[MeSH Terms] OR "plasmodium ovale"[MeSH Terms] OR "malaria, vivax"[MeSH Terms]) AND ("pregnant women"[MeSH Terms] OR "pregnant women"[MeSH Terms])). Hits: 4,344

#2 AND #3 ("malaria, falciparum"[MeSH Terms] OR "malaria, falciparum"[MeSH Terms] OR "plasmodium ovale"[MeSH Terms] OR "malaria, vivax"[MeSH Terms]) AND ("pregnant women"[MeSH Terms] OR "pregnant women"[MeSH Terms]). Hits: 51

"malaria, falciparum"[MeSH Terms] OR "malaria, falciparum"[MeSH Terms] OR "plasmodium ovale"[MeSH Terms] OR "malaria, vivax"[MeSH Terms]. Hits: 15642

"malaria, falciparum"[MeSH Terms] OR "malaria, falciparum"[MeSH Terms] OR "plasmodium ovale"[MeSH Terms] OR "malaria, vivax"[MeSH Terms]. Hits: 24,767

"malaria in pregnancy"[Title/Abstract] OR "placental malaria"[Title/Abstract] OR "antenatal malaria"[Title/Abstract] OR ("pregnancy"[Title/Abstract] AND "Malaria"[Title/Abstract]) OR ("obstetric"[Title/Abstract] AND "Malaria"[Title/Abstract]) OR (("obstetric"[All Fields] OR "obstetrically"[All Fields] OR "obstetrics"[MeSH Terms] OR "obstetrics"[All Fields] OR

"obstetrical"[All Fields]) AND "Malaria"[Title/Abstract]) OR ("Plasmodium"[Title/Abstract] AND "pregnancy"[Title/Abstract]). Hits: 4,336

Final strategy in Medline: 2,161 hits

((("malaria in pregnancy"[Title/Abstract] OR "placental malaria"[Title/Abstract] OR "antenatal malaria"[Title/Abstract] OR ("pregnancy"[Title/Abstract] AND "Malaria"[Title/Abstract]) OR ("obstetric"[Title/Abstract] AND "Malaria"[Title/Abstract]) OR (("obstetric"[All Fields] OR "obstetrically"[All Fields] OR "obstetrics"[MeSH Terms] OR "obstetrics"[All Fields] OR "obstetrical"[All Fields]) AND "Malaria"[Title/Abstract]) OR ("Plasmodium"[Title/Abstract] AND "pregnancy"[Title/Abstract]) OR (("malaria, falciparum"[MeSH Terms] OR "malaria, falciparum"[MeSH Terms] OR "plasmodium ovale"[MeSH Terms] OR "malaria, vivax"[MeSH Terms]) AND ("pregnant women"[MeSH Terms] OR "pregnant women"[MeSH Terms]))) AND ("prevalence"[Title/Abstract] OR "incidence"[Title/Abstract] OR "burden"[Title/Abstract] OR "epidemiology"[Title/Abstract] OR "risk"[Title/Abstract] OR "rate"[Title/Abstract] OR "seroprevalence"[Title/Abstract] OR "prevalence"[MeSH Terms] OR "incidence"[MeSH Terms] OR "cost of illness"[MeSH Terms] OR "epidemiology"[MeSH Terms])) OR (("malaria in pregnancy"[Title/Abstract] OR "placental malaria"[Title/Abstract] OR "antenatal malaria"[Title/Abstract] OR ("pregnancy"[Title/Abstract] AND "Malaria"[Title/Abstract]) OR ("obstetric"[Title/Abstract] AND "Malaria"[Title/Abstract]) OR (("obstetric"[All Fields] OR "obstetrically"[All Fields] OR "obstetrics"[MeSH Terms] OR "obstetrics"[All Fields] OR "obstetrical"[All Fields]) AND "Malaria"[Title/Abstract]) OR ("Plasmodium"[Title/Abstract] AND "pregnancy"[Title/Abstract]) OR (("malaria, falciparum"[MeSH Terms] OR "malaria, falciparum"[MeSH Terms] OR "plasmodium ovale"[MeSH Terms] OR "malaria, vivax"[MeSH Terms]) AND ("pregnant women"[MeSH Terms] OR "pregnant women"[MeSH Terms]))) AND ("anemia, iron deficiency"[MeSH Terms] OR ("anaemia"[Title/Abstract] OR "anemia"[Title/Abstract]) OR "cerebral malaria"[Title/Abstract] OR "malaria, cerebral"[MeSH Terms] OR "malaria, cerebral"[MeSH Terms] OR "maternal mortality"[MeSH Terms] OR "maternal death"[Title/Abstract] OR "mortality"[Title/Abstract] OR "severe malaria"[Title/Abstract] OR "hyperparasitaemia"[Title/Abstract] OR "hyperparasitemia"[Title/Abstract] OR "maternal complication"[Title/Abstract] OR "pregnancy complications"[MeSH Terms])) OR (("malaria in pregnancy"[Title/Abstract] OR "placental malaria"[Title/Abstract] OR "antenatal malaria"[Title/Abstract] OR ("pregnancy"[Title/Abstract] AND "Malaria"[Title/Abstract]) OR ("obstetric"[Title/Abstract] AND "Malaria"[Title/Abstract]) OR (("obstetric"[All Fields] OR "obstetrically"[All Fields] OR "obstetrics"[MeSH Terms] OR "obstetrics"[All Fields] OR "obstetrical"[All Fields]) AND "Malaria"[Title/Abstract]) OR ("Plasmodium"[Title/Abstract] AND "pregnancy"[Title/Abstract]) OR (("malaria, falciparum"[MeSH Terms] OR "malaria, falciparum"[MeSH Terms] OR "plasmodium ovale"[MeSH Terms] OR "malaria, vivax"[MeSH Terms]) AND ("pregnant women"[MeSH Terms] OR "pregnant women"[MeSH Terms]))) AND ("abortion, spontaneous"[MeSH Terms] OR "abortion, spontaneous"[MeSH Terms] OR "stillbirth"[MeSH Terms] OR "premature birth"[MeSH Terms] OR "premature birth"[MeSH Terms] OR "fetal growth retardation"[MeSH Terms] OR ((("retard"[All Fields] OR "retardance"[All Fields] OR "retardances"[All Fields] OR "retardation"[All Fields] OR "retardations"[All Fields] OR "retarded"[All Fields] OR "retarder"[All Fields] OR "retarders"[All Fields] OR "retarding"[All Fields] OR "retards"[All Fields]) AND ("intrauterin"[All Fields] OR "intrauterine"[All Fields])) AND

"growth"[MeSH Terms]) OR "infant, low birth weight"[MeSH Terms] OR "infant mortality"[MeSH Terms] OR "infant mortality"[MeSH Terms] OR ("abortion"[Title/Abstract] OR "miscarriage"[Title/Abstract] OR "stillbirth"[Title/Abstract] OR "premature birth"[Title/Abstract] OR "preterm"[Title/Abstract] OR "intrauterine growth retardation"[Title/Abstract] OR "IUGR"[Title/Abstract] OR "congenital malaria"[Title/Abstract] OR "mortality"[Title/Abstract] OR "death"[Title/Abstract] OR "complication"[Title/Abstract] OR ("sequealae"[All Fields] AND "OR"[Title/Abstract])) OR ("anemia"[Title/Abstract] OR "anaemia"[Title/Abstract]))) OR (("malaria in pregnancy"[Title/Abstract] OR "placental malaria"[Title/Abstract] OR "antenatal malaria"[Title/Abstract] OR ("pregnancy"[Title/Abstract] AND "Malaria"[Title/Abstract]) OR ("obstetric"[Title/Abstract] AND "Malaria"[Title/Abstract]) OR (("obstetric"[All Fields] OR "obstetrically"[All Fields] OR "obstetrics"[MeSH Terms] OR "obstetrics"[All Fields] OR "obstetrical"[All Fields]) AND "Malaria"[Title/Abstract]) OR ("Plasmodium"[Title/Abstract] AND "pregnancy"[Title/Abstract]) OR (("malaria, falciparum"[MeSH Terms] OR "malaria, falciparum"[MeSH Terms] OR "plasmodium ovale"[MeSH Terms] OR "malaria, vivax"[MeSH Terms]) AND ("pregnant women"[MeSH Terms] OR "pregnant women"[MeSH Terms]))) AND ("risk factors"[MeSH Terms] OR ("risk factor"[Title/Abstract] OR "risk group"[Title/Abstract]) OR ("parity"[Title/Abstract] OR "gravidity"[Title/Abstract] OR "age"[Title/Abstract] OR "transmission intensity"[Title/Abstract] OR "endemicity"[Title/Abstract] OR "season"[Title/Abstract] OR "prophylaxis"[Title/Abstract] OR "bednet"[Title/Abstract] OR "socioeconomic"[Title/Abstract]) OR "parity"[MeSH Terms] OR "gravidity"[MeSH Terms] OR "insecticide treated bednets"[MeSH Terms] OR "endemic diseases"[MeSH Terms] OR "social class"[MeSH Terms] OR ("ethnology"[MeSH Terms] OR "ethnicity"[MeSH Terms])))) AND (2013:2024[pdat])

SCOPUS

(((((((Malaria in pregnancy[Title/Abstract]) OR (placental malaria[Title/Abstract])) OR (antenatal malaria[Title/Abstract])) OR ((pregnancy[Title/Abstract] AND malaria[Title/Abstract]))) OR ((obstetric[Title/Abstract] AND malaria[Title/Abstract]))) OR (obstetric malaria[Title/Abstract])) OR ((Plasmodium[Title/Abstract] AND pregnancy[Title/Abstract]))) **2013-March 2025 (2,922 hits)**

Embase **2013 to March 2025 (2,269 hits)**

(((('anemia':ab,kw,ti OR 'cerebral malaria':ab,kw,ti OR 'maternal mortality':ab,kw,ti OR 'maternal death':ab,kw,ti OR 'severe malaria':ab,kw,ti OR 'hyperparasitemia':ab,kw,ti OR 'pregnancy complication':ab,kw,ti) OR ('abortion':ab,kw,ti OR 'spontaneous abortion':ab,kw,ti OR 'stillbirth':ab,kw,ti OR 'premature labor':ab,kw,ti OR 'prematurity':ab,kw,ti OR 'intrauterine growth retardation':ab,kw,ti OR 'child death':kw,ab,ti OR 'infant mortality':ab,kw,ti OR 'newborn mortality':ab,kw,ti OR 'low birth weight':ab,kw,ti) OR ('risk factor':ab,kw,ti OR 'high risk population':ab,kw,ti OR 'parity':kw,ab,ti OR 'age':ab,kw,ti OR 'endemic disease':ab,kw,ti OR endemicity:ab,kw,ti OR 'season':ab,kw,ti OR prophylaxis:ab,kw,ti OR 'chemoprophylaxis':ab,kw,ti OR 'insecticide treated net':ab,kw,ti) OR ('prevalence':ab,kw,ti OR 'incidence':kw,ti,ab OR 'epidemiology':ab,ti,kw OR 'disease burden':ab,kw,ti OR 'risk':ab,kw,ti OR 'frequency':ab,kw,ti)) AND ('placenta* malaria':ab,kw,ti OR 'malaria in pregnancy':ab,kw,ti

OR (malaria:ab,ti AND pregnancy:ab,ti)) AND (2013:py OR 2014:py OR 2015:py OR 2016:py OR 2017:py OR 2018:py OR 2019:py OR 2020:py OR 2021:py OR 2022:py OR 2023:py OR 2024:py))

Table of excluded full text screened studies

Author	Reason for exclusion	Doi
**Accrombessi et al (2015)**	Study before 2013	10.1371/journal.pone.0129510
**Adam et al (2017)**	Study before 2013	10.1371/journal.pone.0182394
**Agudelo et al (2013)**	Study before 2013	10.1186/1475-2875-12-341
**Agudelo et al (2013)**	Study before 2013	10.1186/1475-2875-12-341.
**Ahenkorah et al (2019)**	No relevant outcome	10.1155/2019/2094560
**Ahmed et al (2014)**	Study before 2013	10.1186/1475-2875-13-232
**Ahmed et al (2014)**	Study before 2013	10.1186/1475-2875-13-232.
**Akinnawo et al (2022)**	Study before 2013	10.1186/s12936-022-04252-0
**Alim et al (2015)**	Study before 2013	10.1186/s13000-015-0275-3
**Alonso et al (2021)**	Study before 2013	10.1016/j.jinf.2021.02.024
**Alvarado et al (2015)**	Study before 2013	NR
**Arango et al (2013)**	Study before 2013	10.4269/ajtmh.12-0669
**Arinaitwe et al (2013)**	Study before 2013	10.1371/journal.pone.0073073
**Asante et al (2013)**	Study before 2013	10.1093/infdis/jit366
**Atekega et al (2020)**	Replicated analysis	10.1093/infdis/jiaa156
**Atemba et al (2013)**	Study before 2013	NR
**Atwell et al (2016)**	Study before 2013	10.1093/infdis/jiv401
**Awine et al (2016)**	Study before 2013	10.1186/s12936-016-1094-z
**Azizi et al (2018)**	No relevant outcome	10.1186/s12884-018-1744-y
**Barry et al (2013)**	Study before 2013	NR

**Barsosio et al (2024)**	Wrong study design	10.1016/S0140-6736(23)02631-4
**Bassey et al (2015)**	Study before 2013	10.4103/1119-3077.146975
**Batran et al (2013)**	Study before 2013	10.1186/1746-1596-8-189
**Bedu-Addo et al (2014)**	Study before 2013	10.1186/1475-2875-13-289
**Bihoun et al (2022)**	Study before 2013	10.1186/s12884-022-04568-4
**Blay et al (2013)**	No relevant outcome	NR
**Blay et al (2015)**	No relevant outcome	NR
**Borgella et al (2013)**	Study before 2013	10.1371/journal.pone.0080624
**Bouaziz et al (2018)**	Study before 2013	10.1093/cid/cix899
**Boudova et al (2016)**	No relevant outcome	10.1093/ofid/ofw194.55
**Boudova et al (2015)**	Wrong study design	10.1093/ofid/ofv133.1160
**BoudovÃ et al (2017)**	Study before 2013	10.1093/infdis/jix372
**BoudovÃ et al (2014)**	Study before 2013	10.1186/1475-2875-13-506
**Bouyou-Akotet et al (2016)**	Study before 2013	10.1093/trstmh/trw034
**Brickley et al (2015)**	Study before 2013	NR
**Buchwald et al (2022)**	Study before 2013	NR
**Cardona-Arias et al (2022)**	Study before 2013	10.1371/journal.pone.0263451
**Cardona-Arias et al (2022)**	Study before 2013	10.1371/journal.pone.0268949
**Cardona-Arias et al (2021)**	Wrong study design	10.1371/journal.pone.0255028
**Cohee et al (2014)**	Study before 2013	10.1186/1475-2875-13-274
**Cutts et al (2020)**	Wrong study design	10.1186/s12916-019-1467-6
**Dalapati et al (2018)**	Wrong study design	10.1096/fj.201701472R
**Darling et al (2017)**	Study before 2013	10.4269/ajtmh.16-0599
**Das et al (2024)**	Wrong study design	10.1017/S0950268824000177

**Das et al (2024)**	Wrong study design	10.1017/S0950268824000177
**Datta et al (2017)**	No relevant outcome	10.7860/JCDR/2017/23051.9195
**De Beaudrap et al (2013)**	Study before 2013	10.1186/1475-2875-12-139
**Denoeud-Ndam et al (2014)**	Study before 2013	10.1097/QAI.0000000000000058
**Dobaño et al (2020)**	Study before 2013	10.1016/j.cyto.2019.154818
**Doritchamou et al (2018)**	Study before 2013	10.1371/journal.pntd.0006279
**Doumbo et al (2013)**	Study before 2013	10.1007/s13149-013-0301-1
**Elhassan et al (2015)**	Study before 2013	10.1111/tmi.12574
**Elhassan et al (2015)**	Study before 2013	10.1111/tmi.12574
**Elphinstone et al (2019)**	Study before 2013	10.1371/journal.pmed.1002914
**Feleke et al (2020)**	No relevant outcome	10.1186/s12936-020-3152-9
**Gavina et al (2018)**	No relevant outcome	10.1128/IAI.00797-17
**Gaw et al (2018)**	Wrong study design	NR
**Gies et al (2013)**	Study before 2013	10.1111/tmi.12163
**Godwin et al (2022)**	No relevant outcome	10.1177/20499361221122620
**Gutman et al (2021)**	Wrong study design	10.1016/j.eclinm.2021.101160
**Gutman et al (2013)**	Study before 2013	10.1093/infdis/jit276
**Gutman et al (2021)**	Wrong study design	10.1016/j.eclinm.2021.101160
**Harrington et al (2013)**	Study before 2013	10.1371/journal.pone.0056183
**Hughes et al (2020)**	No relevant outcome	10.1128/AAC.01013-20
**Izuka et al (2017)**	Study before 2013	10.4103/1119-3077.180077
**Jaiberth Antonio Cardona-Arias (2021)**	Wrong study design	10.1371/journal.pone.0255028
**Kabyemela et al (2021)**	Wrong outcome	10.3389/fimmu.2021.624136

**Kakuru et al (2019)**	Wrong study design	10.1186/s12936-019-2943-3
**Kakuru et al (2016)**	Study before 2013	NR
**Kalilani-Phiri et al (2013)**	Study before 2013	10.1371/journal.pone.0074643
**Kalinjuma et al (2020)**	Study before 2013	10.1186/s12879-020-05521-6
**Kapisi et al (2016)**	Replicated analysis	10.4269/ajtmh.abstract2016
**Kashif et al (2013)**	Study before 2013	10.1186/1746-1596-8-59
**Kawahara et al (2019)**	Wrong study design	10.1074/mcp.RA118.000907
**Koladjo et al (2022)**	No relevant outcome	10.1093/infdis/jiac012
**Kuepfer et al (2019)**	Study before 2013	10.1136/bmjgh-2019-001399
**Laar et al (2013)**	No relevant outcome	doi:10.1186/1756-0500-6-350
**Lawford et al (2021)**	No relevant outcome	10.1016/j.ijid.2021.07.037
**Liu et al (2016)**	Study before 2013	10.4269/ajtmh.16-0356
**Lloyd et al (2018)**	Study before 2013	10.1128/IAI.00166-18
**Lopez et al (2017)**	No relevant outcome	10.3855/jidc.9182
**Lufele et al (2017)**	Study before 2013	10.1186/s12936-017-2077-4
**Lwamulungi et al (2023)**	No relevant outcome	10.1371/journal.pone.0291172
**Lybbert et al (2016)**	Wrong study design	10.1038/srep24508
**Madanitsa et al (2016)**	Study before 2013	10.1371/journal.pmed.1002124
**Mahamar et al (2021)**	No relevant outcome	10.1093/cid/ciab301
**Mathieu et al (2023)**	No relevant outcome	10.1016/j.eurox.2023.100190
**Mayor et al (2015)**	Study before 2013	10.1056/NEJMoa1406459
**Mbachu et al (2018)**	Duplicate	10.1002/ijgo.12582
**McDonald et al (2018)**	No relevant outcome	10.1126/scitranslmed.aan6007
**McLean et al (2021)**	Study before 2013	10.3389/fimmu.2021.644563

**Megnekou et al (2015)**	Study before 2013	10.1016/j.actatropica.2014.10.007
**Mens et al (2017)**	Wrong study design	NR
**Mikobi et al (2022)**	No relevant outcome	10.1371/journal.pone.0275115
**Mikomangwa et al (2019)**	No relevant outcome	10.1186/s12884-019-2397-1
**Mlugu et al (2020)**	Duplicate	10.3390/pathogens9030207
**Mohamed et al (2013)**	Study before 2013	doi:10.4269/ajtmh.16-0599
**Moore et al (2017)**	Wrong study design	10.1016/S2214-109X(17)30340-6
**Mosha et al (2014)**	Study before 2013	10.1111/tmi.12349
**Mugabe et al (2020)**	Location and study period unknown	NR
**Mutuku et al (2014)**	No relevant outcome	NR
**Nadam et al (2015)**	Study before 2013	10.1186/s12916-017-0893-6.
**Ndam et al (2017)**	Study before 2013	10.1186/s12916-017-0893-6
**Ndeserua et al (2015)**	Study before 2013	10.4314/ahs.v15i3.15
**Nuridiati et al (2018)**	Irretrievable	Conference abstract
**Nwali et al (2014)**	Study before 2013	10.4103/0300-1652.132063
**Obiri et al (2020)**	No relevant outcome	10.1038/s41598-020-64736-4
**Obiri et al (2019)**	No relevant outcome	10.4269/ajtmh.abstract2019
**Omer et al (2020)**	Wrong study design	10.1007/s40475-020-00214-1
**Omer et al (2017)**	Study before 2013	10.1186/s12936-017-2028-0
**Omer et al (2020)**	Study before 2013	10.1093/tropej/fmz083
**Omer et al (2013)**	Study before 2013	10.1111/tmi.12163
**Omer et al (2021)**	Study before 2013	10.1186/s12936-021-03580-x
**Omoniyi-Esan et al (2014)**	Wrong study design	NR
**Onoja et al (2023)**	Wrong study design	10.4103/0972-9062.374243

**Opi et al (2021)**	Study before 2013	10.1186/s12916-021-02061-x
**Oraneli et al (2013)**	Study before 2013	NR
**Ouédraogo et al (2019)**	Study before 2013	10.11604/pamj.2019.34.30.20013
**Ozarslan et al (2019)**	Wrong study design	10.1177/1933719119834079
**Park et al (2020)**	Wrong study design	10.1093/infdis/jiaa139
**Patel et al (2016)**	Wrong study design	10.4269/ajtmh.abstract2016
**Patel et al (2017)**	Wrong outcome	10.1038/s41598-017-04737-y
**Patel et al (2016)**	Study before 2013	10.4269/ajtmh.15-0672
**Plotkin et al (2014)**	Study before 2013	10.4269/ajtmh.13-0586
**Pons Duran et al (2022)**	Study before 2013	10.1371/journal.pmed.1004084
**Quanquin et al (2020)**	No relevant outcome	10.1186/s12936-020-03351-0
**Randall et al (2015)**	No relevant outcome	NR
**Sadoh et al (2018)**	No relevant outcome	10.1097/QAI.0000000000001786.
**Saito et al (2020)**	Wrong study design	10.1186/s12916-020-01592-z
**Schultz et al (2018)**	Study before 2013	NR
**Simon et al (2021)**	No relevant outcome	10.1093/infdis/jiab275
**Singh et al (2020)**	Study before 2013	10.1155/2020/6163487
**Stanisic et al (2015)**	Study before 2013	10.1093/trstmh/trv019
**Tagbor et al (2015)**	Study before 2013	10.1371/journal.pone.0132247
**Tahita et al (2023)**	No relevant outcome	10.1136/bmjgh-2023-EDC.281
**Tahita et al (2022)**	Wrong study design	10.1186/s40814-022-01181-2
**Taylor et al (2017)**	Wrong study design	10.1016/S2214-109X(17)30378-9
**Tonga et al (2013)**	Study before 2013	10.1371/journal.pone.0065876
**Toure et al (2014)**	Study before 2013	10.1186/s13071-014-0495-5

**Ukaga et al (2016)**	Unknown sample size	NR
**Unger et al (2015)**	Study before 2013	10.1186/s12916-014-0258-3
**Unger et al (2022)**	Study before 2013	10.1186/s12936-022-04177-8
**Valderramos et al (2014)**	No relevant outcome	10.1016/j.ajog.2013.10.484
**van Eijk et al (2014)**	Wrong study design	10.1016/S2214-109X(23)00194-8
**Vincenz et al (2022)**	Study before 2013	10.1186/s12936-022-04125-6
**Walker et al (2024)**	Wrong study design	10.1016/S2214-109X(14)70256-6
**Weckman et al (2018)**	No relevant outcome	NR
**Yimam et al (2021)**	No relevant outcome	10.1371/journal.pone.0248245
**Yunga et al (2018)**	Study before 2013	DOI:10.1038/s41598-017-18574-6
**Zablon et al (2015)**	No relevant outcome	10.1155/2015/473203

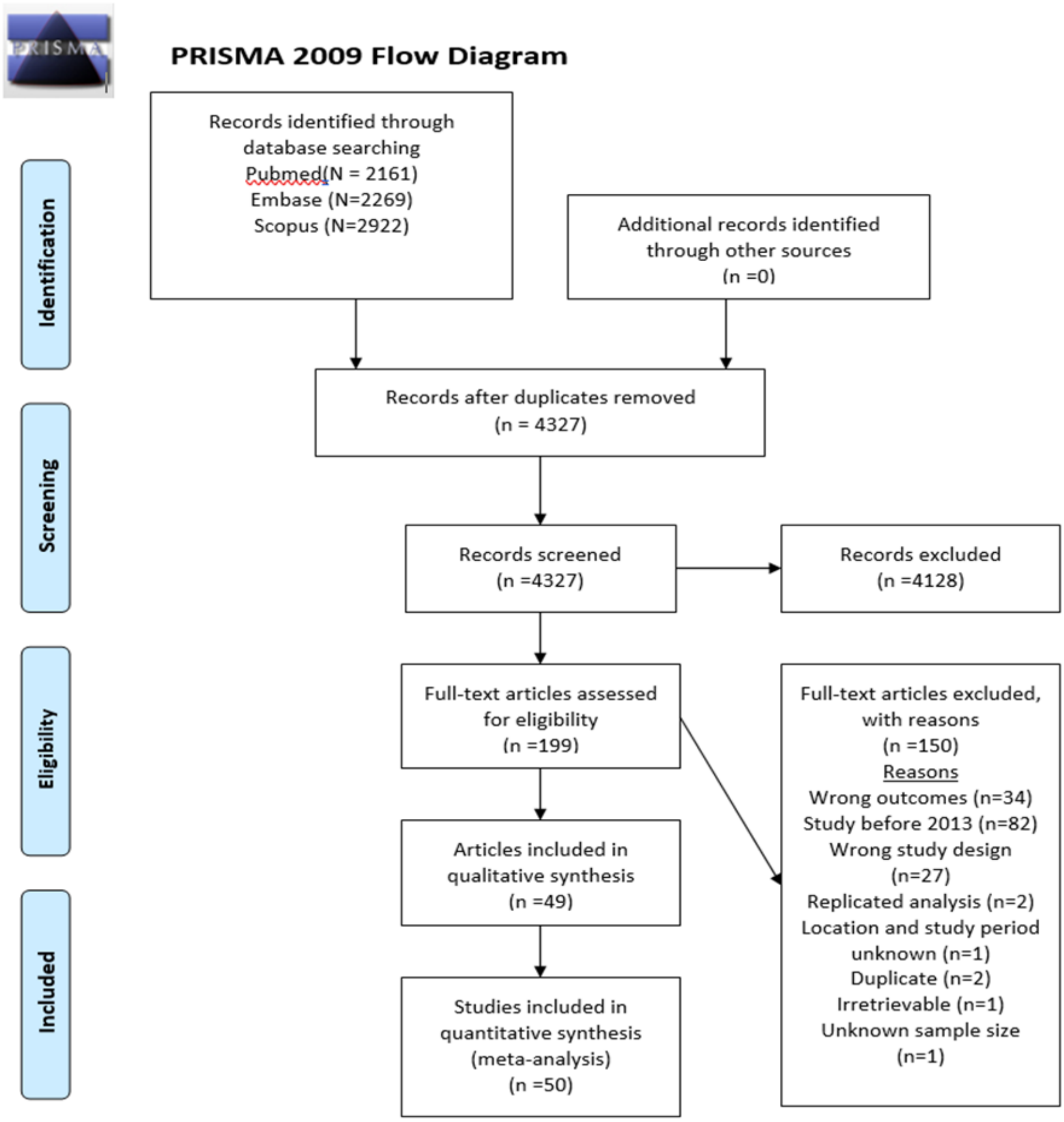

Fig S1 PRISMA flow chart

Risk of bias assessment

The representativeness of the study samples was categorised as follows: truly representative (random sampling or consecutive sampling across two or more sites, or in a national/reference hospital) awarded 2 points; somewhat representative (consecutive sampling at a single site or convenience sampling in a reference/general hospital) awarded 1 point; and not representative (convenience sampling or no description provided) awarded 0 point. For sample size assessment, studies received 1 point if they justified their sample size and 0 points if no justification was provided. Outcome ascertainment was rated based on diagnostic methods: 2 points for studies using the gold standard histological diagnosis, 1 point for other diagnostic methods, and 0 points for those with no diagnostic description.

For prospective cohort studies, given that both arms were combined, the comparability domain was not assessed. Instead, we evaluated the representativeness of the entire sample, including both exposed and unexposed groups. The adequacy of exposure ascertainment and the absence of the outcome at baseline/start of the study were dichotomized as yes (1 point) and no or no information (0 points). Adequate follow-up was defined as complete follow-up or a loss to follow-up rate of less than 20% (1 point), with higher rates of loss to follow-up or insufficient description receiving 0 points.

Summary plot: all outcomes

Cross sectional studies

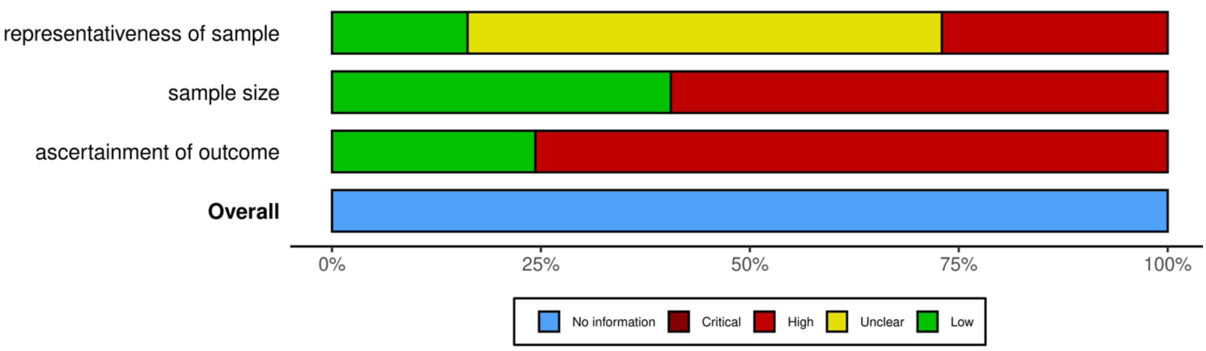

Fig S2 Summary plot for cross sectional studies: all outcomes

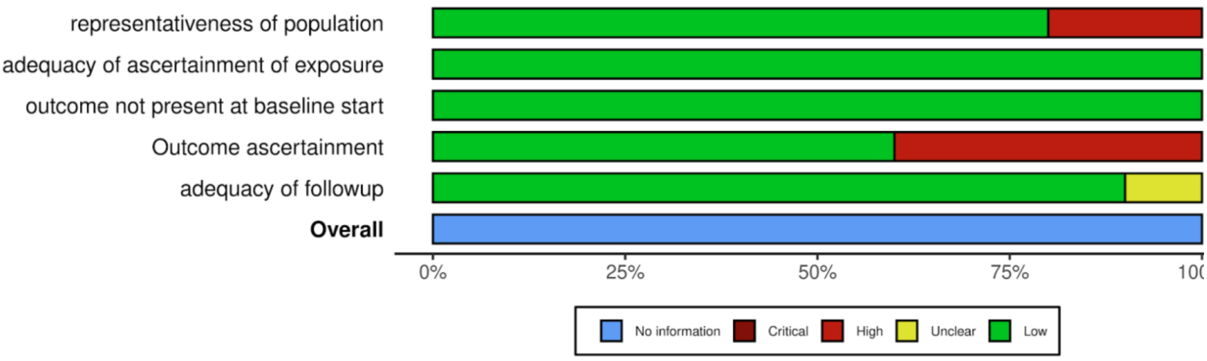

Fig S3 Summary plot for cohort studies: all outcomes

Additional supplementary figures

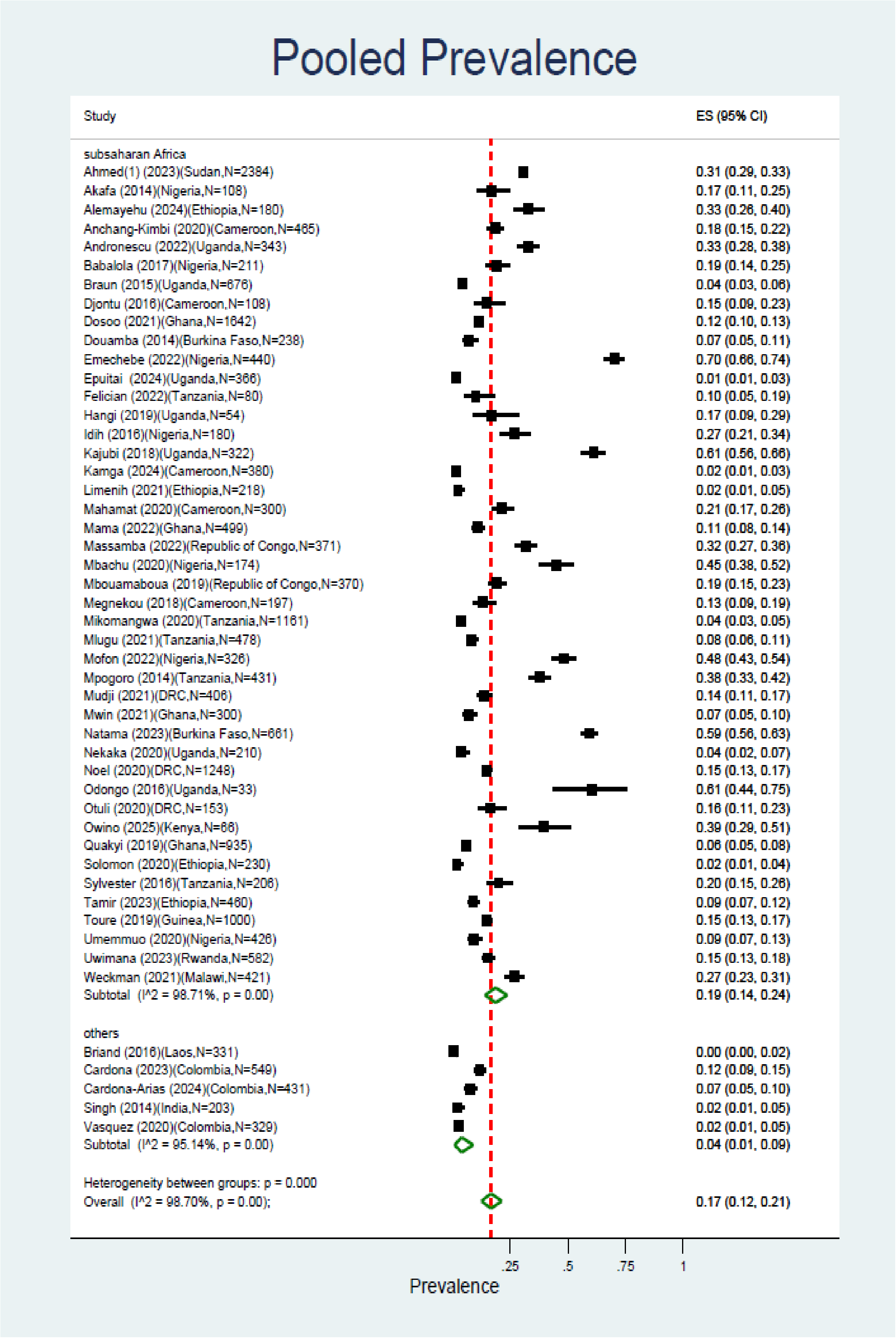

Figure S4 Pooled PM prevalence by region

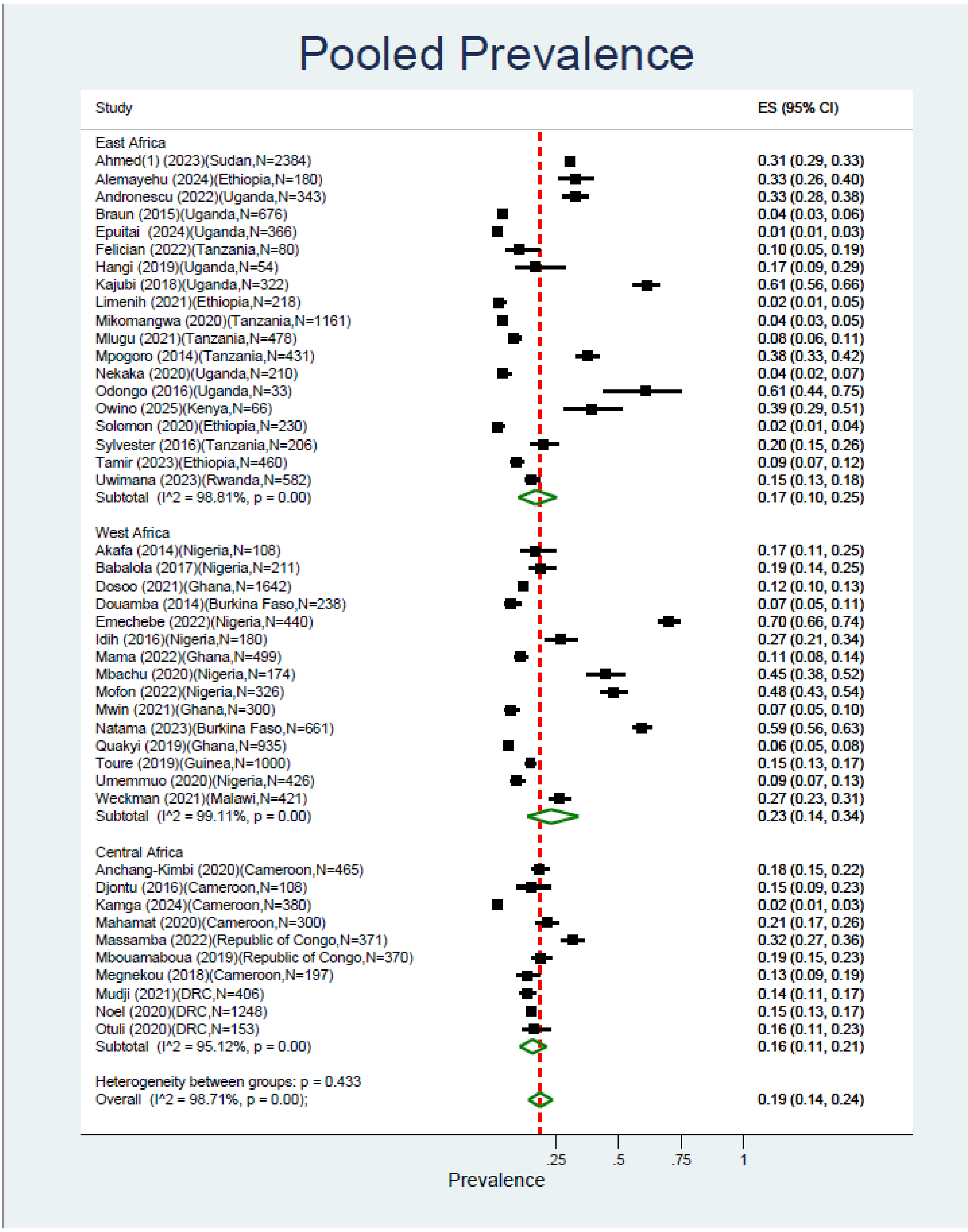

Figure S5 Pooled PM prevalence by region in Sub Saharan Africa

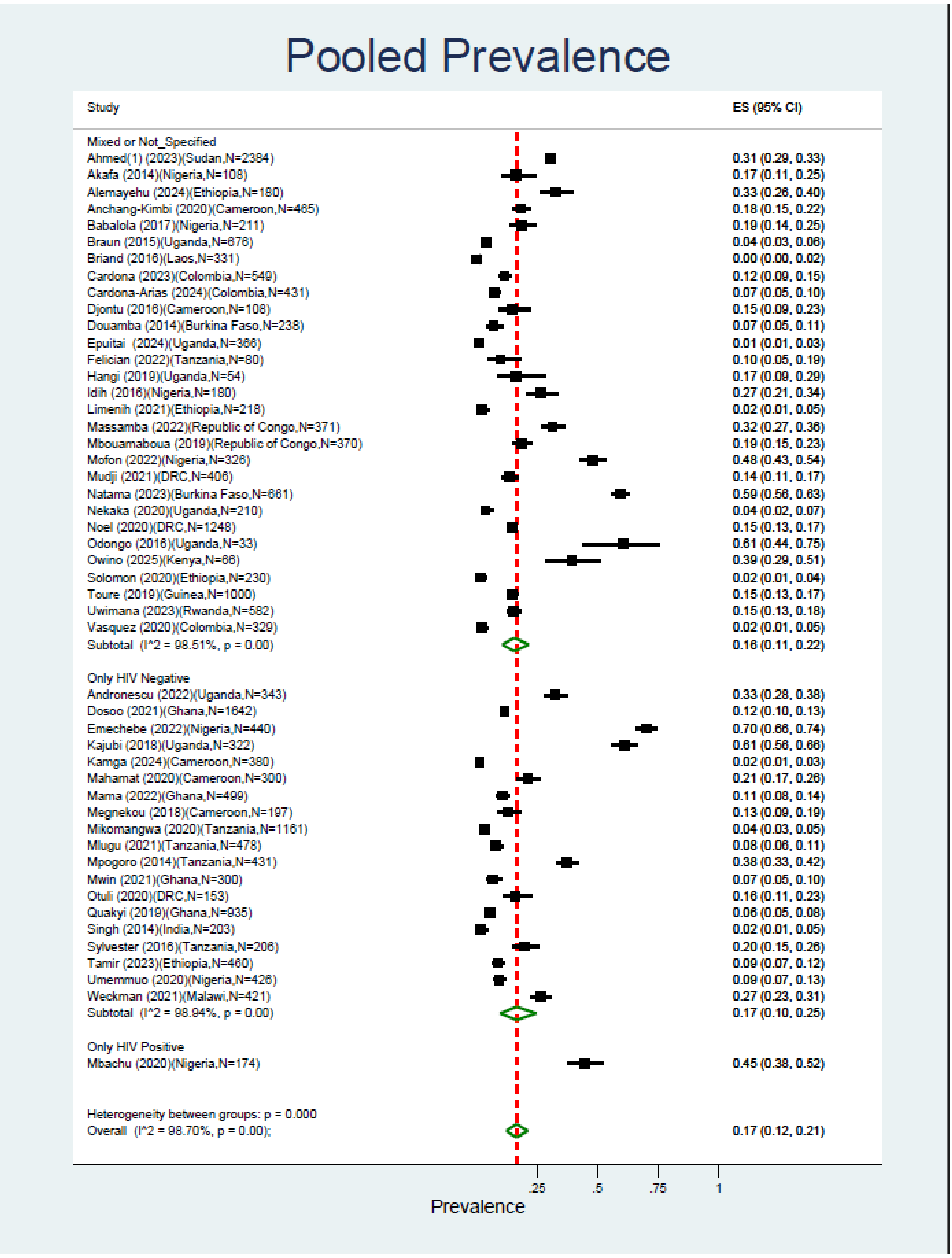

Figure S6 Pooled PM prevalence by HIV status

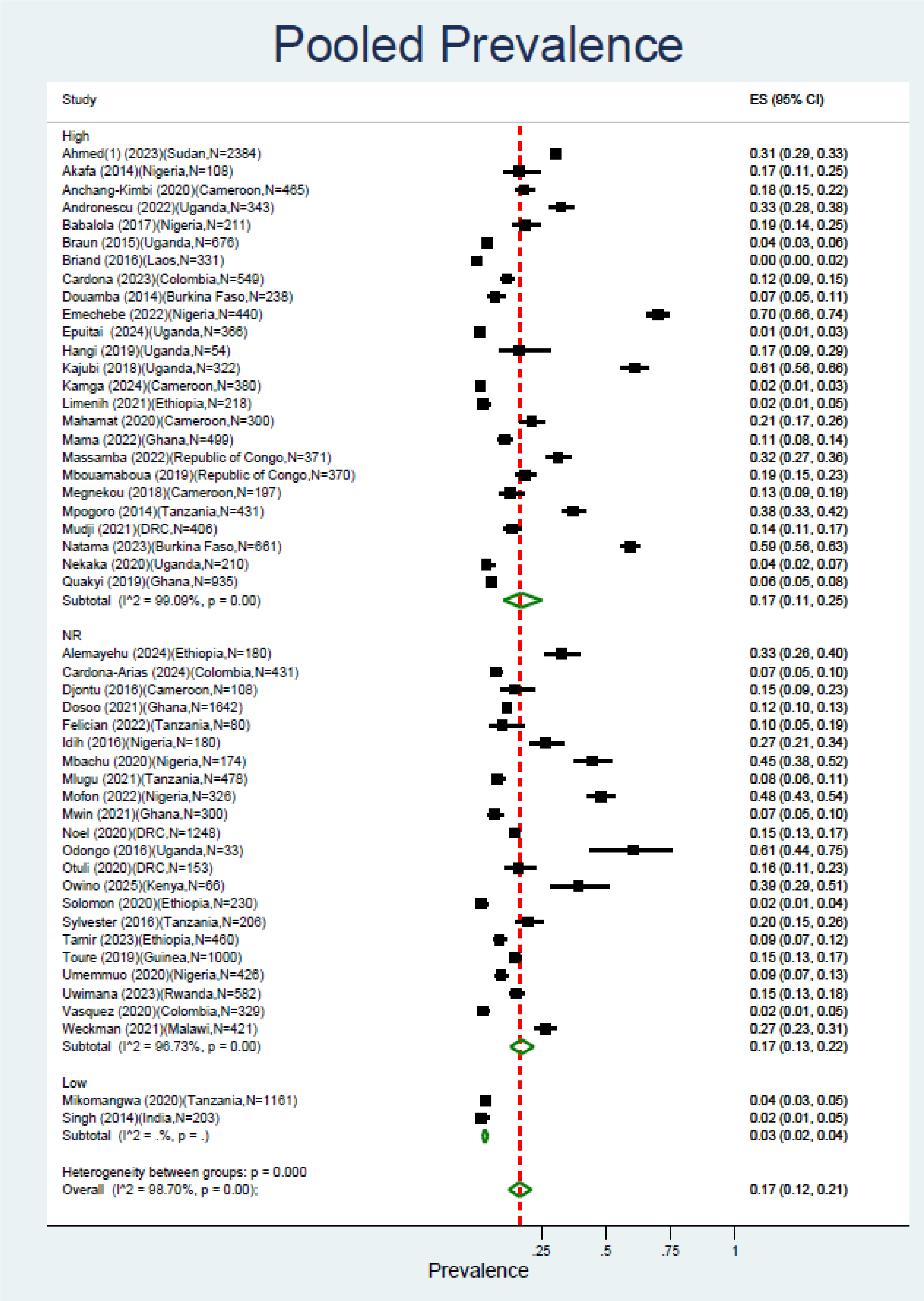

Figure S7 Pooled PM prevalence by malaria transmission setting

Subgroup analysis by histology

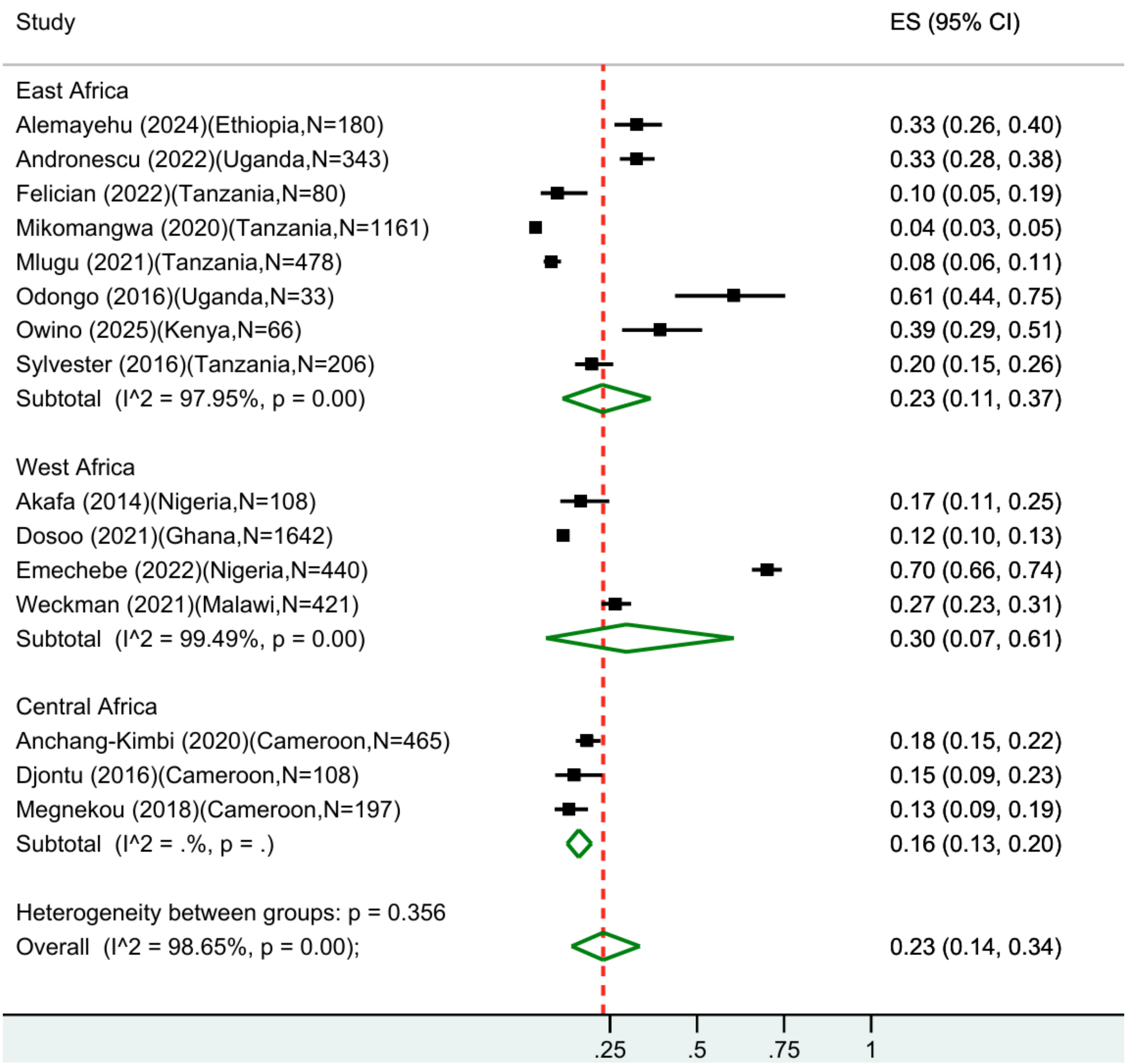

Figure S8 Pooled PM prevalence by region in Sub Saharan Africa.

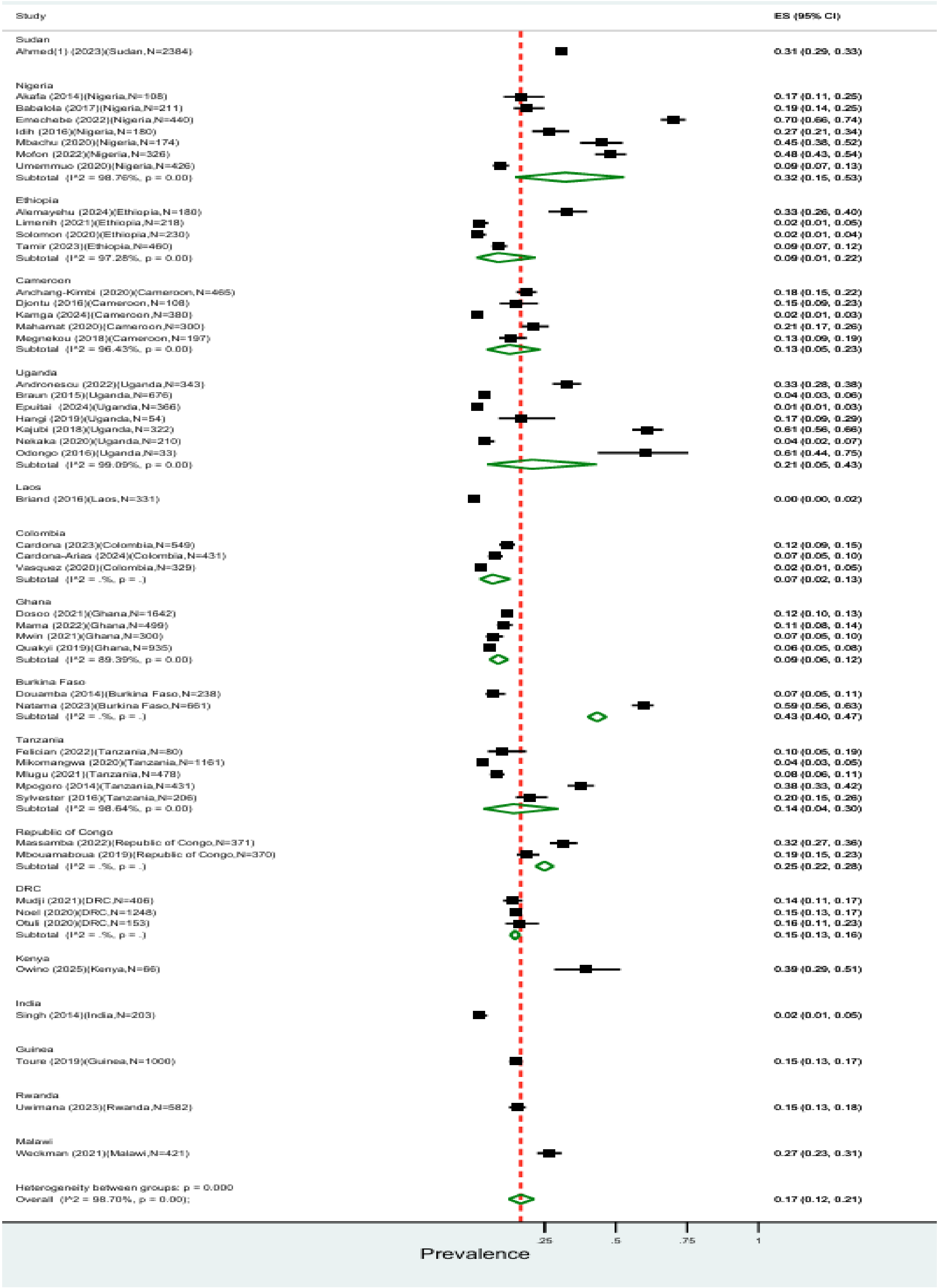

Figure S9 Pooled PM prevalence by country (histology)

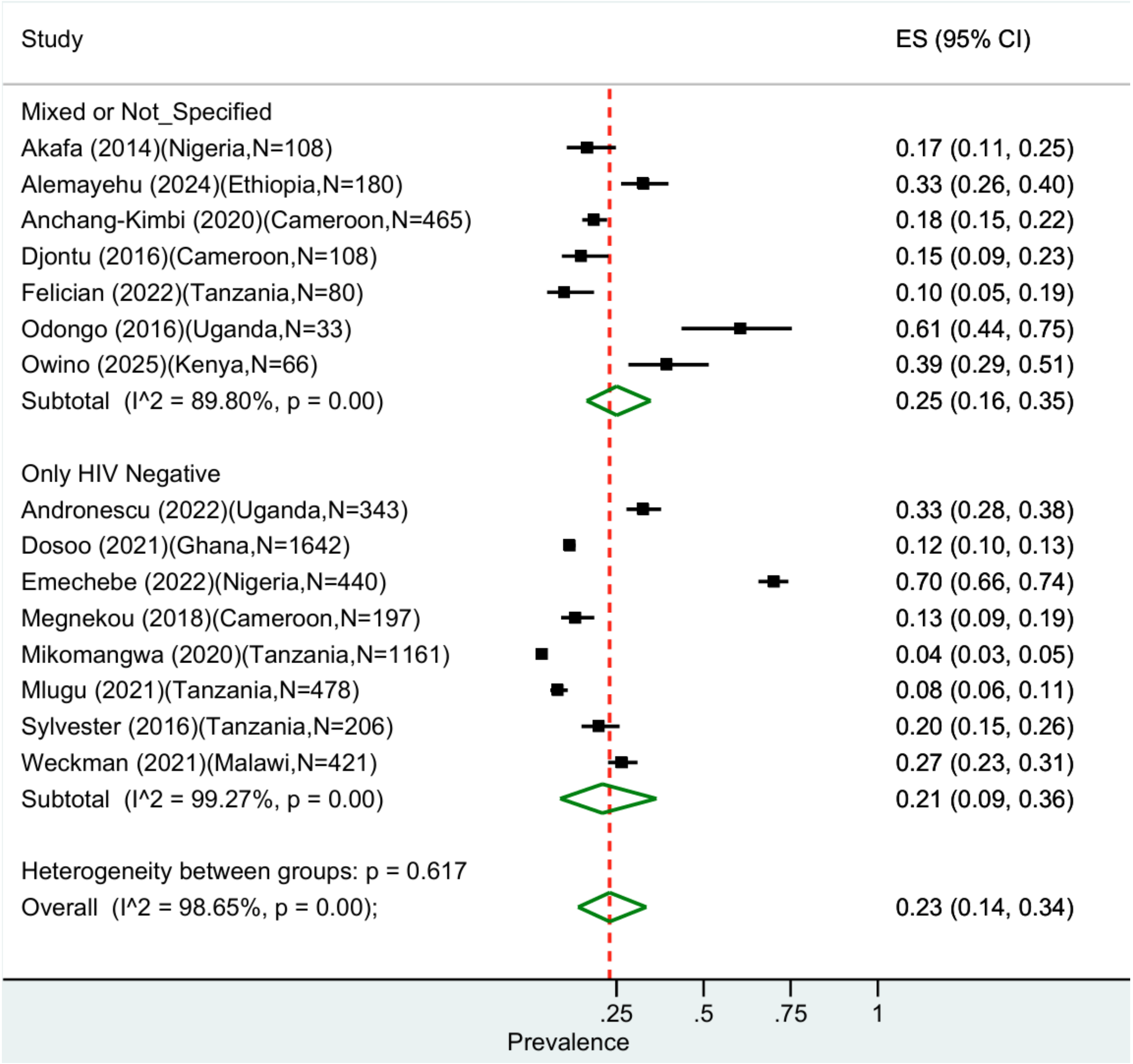

Figure S10 Prevalence by HIV status (histology)

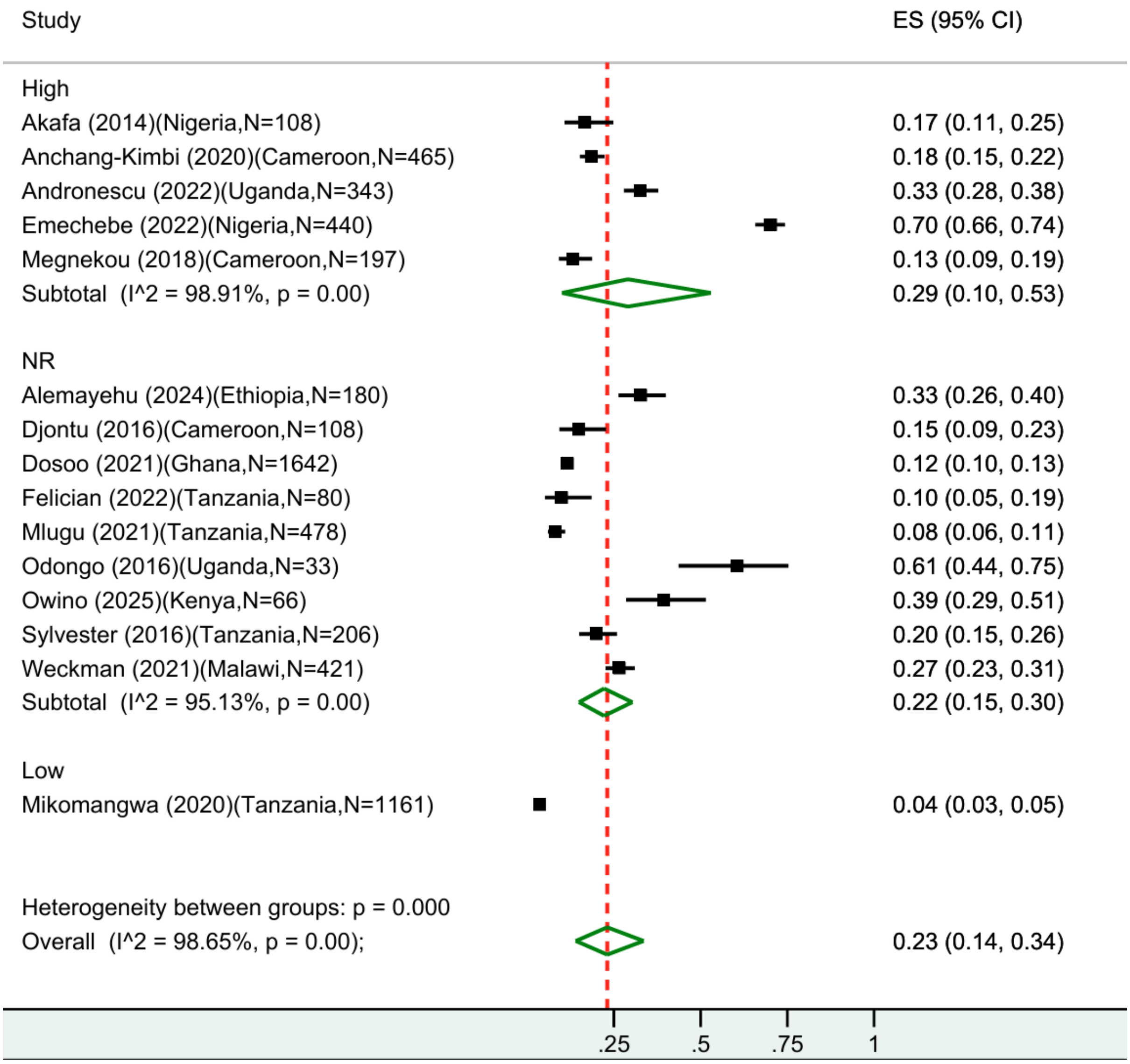

Figure S11 Pooled PM prevalence by transmission setting by histology

Risk factors for PM

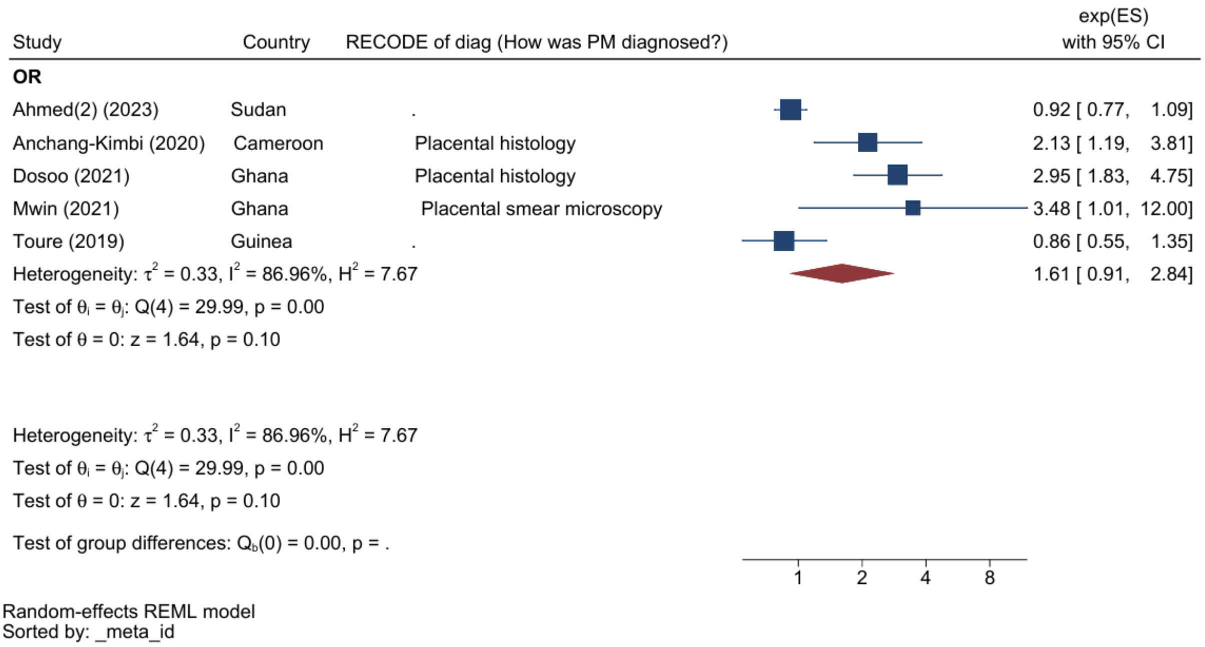

Fig S12 Primigravida compared to multigravida

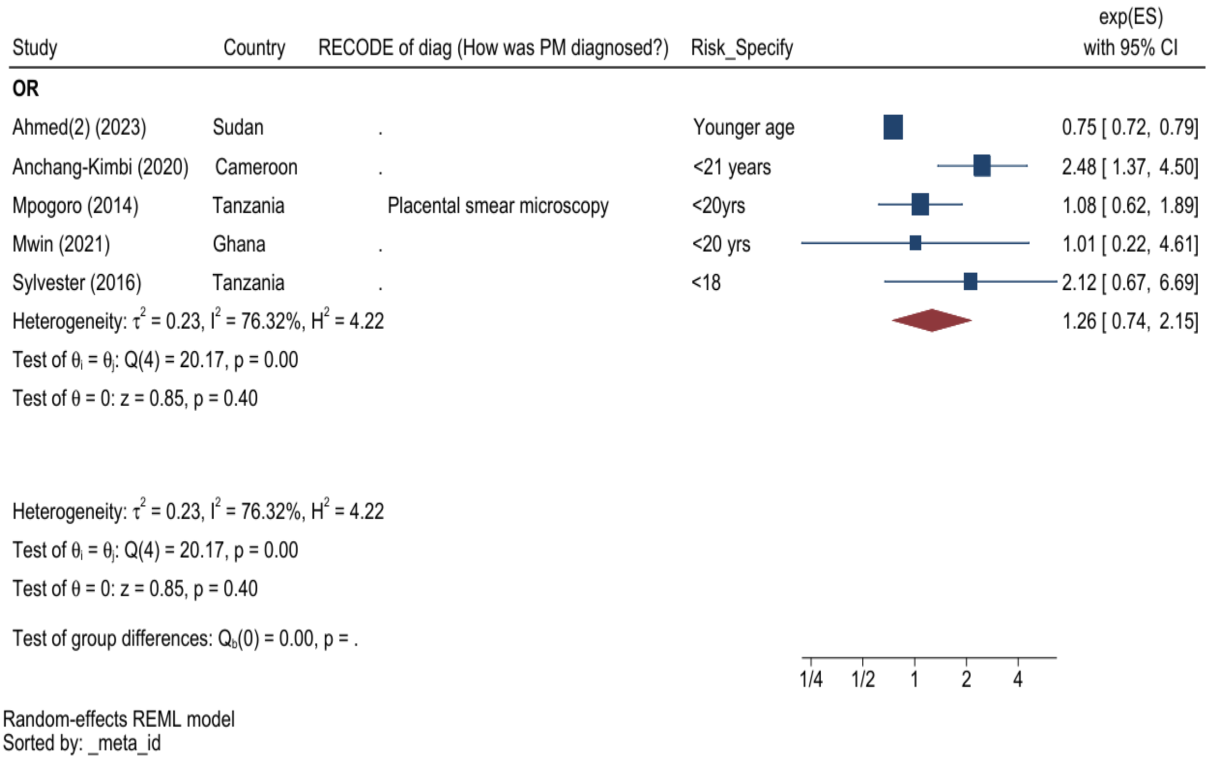

Fig S13 Older age compared to younger age

Foetal outcomes associated with PM

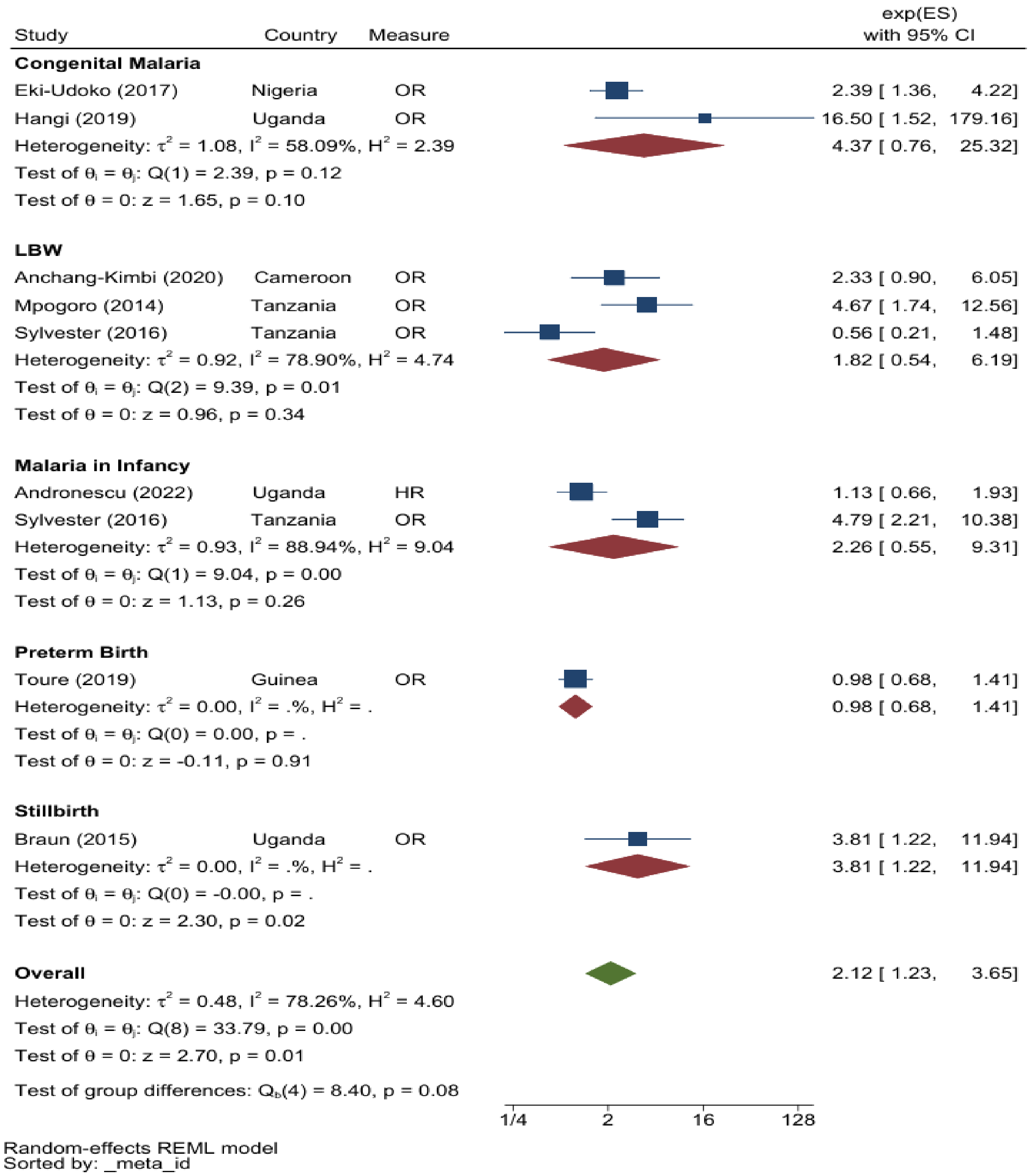

Fig S14 Foetal outcomes

Section and Topic	Item #	Checklist item	Location where item is reported
**TITLE**			
Title	1	Identify the report as a systematic review.	1
**ABSTRACT**			
Abstract	2	See the PRISMA 2020 for Abstracts checklist.	2
**INTRODUCTION**			
Rationale	3	Describe the rationale for the review in the context of existing knowledge.	3
Objectives	4	Provide an explicit statement of the objective(s) or question(s) the review addresses.	5
**METHODS**			
Eligibility criteria	5	Specify the inclusion and exclusion criteria for the review and how studies were grouped for the syntheses.	5-6
Information sources	6	Specify all databases, registers, websites, organisations, reference lists and other sources searched or consulted to identify studies. Specify the date when each source was last searched or consulted.	6
Search strategy	7	Present the full search strategies for all databases, registers and websites, including any filters and limits used.	Annex
Selection process	8	Specify the methods used to decide whether a study met the inclusion criteria of the review, including how many reviewers screened each record and each report retrieved, whether they worked independently, and if applicable, details of automation tools used in the process.	6
Data collection process	9	Specify the methods used to collect data from reports, including how many reviewers collected data from each report, whether they worked independently, any processes for obtaining or confirming data from study investigators, and if applicable, details of automation tools used in the process.	6
Data items	10a	List and define all outcomes for which data were sought. Specify whether all results that were compatible with each outcome domain in each study were sought (e.g. for all measures, time points, analyses), and if not, the methods used to decide which results to collect.	6
	10b	List and define all other variables for which data were sought (e.g. participant and intervention characteristics, funding sources). Describe any assumptions made about any missing or unclear information.	7
Study risk of bias assessment	11	Specify the methods used to assess risk of bias in the included studies, including details of the tool(s) used, how many reviewers assessed each study and whether they worked independently, and if applicable, details of automation tools used in the process.	6 and Annex
Effect measures	12	Specify for each outcome the effect measure(s) (e.g. risk ratio, mean difference) used in the synthesis or presentation of results.	7
Synthesis methods	13a	Describe the processes used to decide which studies were eligible for each synthesis (e.g. tabulating the study intervention characteristics and comparing against the planned groups for each synthesis (item #5)).	6-7
	13b	Describe any methods required to prepare the data for presentation or synthesis, such as handling of missing summary statistics, or data conversions.	7-8
	13c	Describe any methods used to tabulate or visually display results of individual studies and syntheses.	7-8
	13d	Describe any methods used to synthesize results and provide a rationale for the choice(s). If meta-analysis was performed, describe the model(s), method(s) to identify the presence and extent of statistical heterogeneity, and software package(s) used.	7-8

Section and Topic	Item #	Checklist item	Location where item is reported
	13e	Describe any methods used to explore possible causes of heterogeneity among study results (e.g. subgroup analysis, meta-regression).	7-8
	13f	Describe any sensitivity analyses conducted to assess robustness of the synthesized results.	8
Reporting bias assessment	14	Describe any methods used to assess risk of bias due to missing results in a synthesis (arising from reporting biases).	8
Certainty assessment	15	Describe any methods used to assess certainty (or confidence) in the body of evidence for an outcome.	NA
**RESULTS**			
Study selection	16a	Describe the results of the search and selection process, from the number of records identified in the search to the number of studies included in the review, ideally using a flow diagram.	8
	16b	Cite studies that might appear to meet the inclusion criteria, but which were excluded, and explain why they were excluded.	Annex/Prisma Flow chart
Study characteristics	17	Cite each included study and present its characteristics.	8 (table 1)
Risk of bias in studies	18	Present assessments of risk of bias for each included study.	8and 9
Results of individual studies	19	For all outcomes, present, for each study: (a) summary statistics for each group (where appropriate) and (b) an effect estimate and its precision (e.g. confidence/credible interval), ideally using structured tables or plots.	8-11
Results of syntheses	20a	For each synthesis, briefly summarise the characteristics and risk of bias among contributing studies.	8-11
	20b	Present results of all statistical syntheses conducted. If meta-analysis was done, present for each the summary estimate and its precision (e.g. confidence/credible interval) and measures of statistical heterogeneity. If comparing groups, describe the direction of the effect.	8-11
	20c	Present results of all investigations of possible causes of heterogeneity among study results.	8-11
	20d	Present results of all sensitivity analyses conducted to assess the robustness of the synthesized results.	10 and Annex
Reporting biases	21	Present assessments of risk of bias due to missing results (arising from reporting biases) for each synthesis assessed.	10 and Annex
Certainty of evidence	22	Present assessments of certainty (or confidence) in the body of evidence for each outcome assessed.	NA
**DISCUSSION**			
Discussion	23a	Provide a general interpretation of the results in the context of other evidence.	11-13
	23b	Discuss any limitations of the evidence included in the review.	14-15
	23c	Discuss any limitations of the review processes used.	14-15

Section and Topic	Item #	Checklist item	Location where item is reported
	23d	Discuss implications of the results for practice, policy, and future research.	14-15
**OTHER INFORMATION**			
Registration and protocol	24a	Provide registration information for the review, including register name and registration number, or state that the review was not registered.	5
	24b	Indicate where the review protocol can be accessed, or state that a protocol was not prepared.	5
	24c	Describe and explain any amendments to information provided at registration or in the protocol.	NA
Support	25	Describe sources of financial or non-financial support for the review, and the role of the funders or sponsors in the review.	16
Competing interests	26	Declare any competing interests of review authors.	16
Availability of data, code and other materials	27	Report which of the following are publicly available and where they can be found: template data collection forms; data extracted from included studies; data used for all analyses; analytic code; any other materials used in the review.	

Fig S15 PRISMA checklist
